# Genome-wide detection of human intronic AG-gain variants located between splicing branchpoints and canonical splice acceptor sites

**DOI:** 10.1073/pnas.2314225120

**Published:** 2023-11-06

**Authors:** Peng Zhang, Matthieu Chaldebas, Masato Ogishi, Fahd Al Qureshah, Khoren Ponsin, Yi Feng, Darawan Rinchai, Baptiste Milisavljevic, Ji Eun Han, Marcela Moncada-Vélez, Sevgi Keles, Bernd Schröder, Peter D. Stenson, David N. Cooper, Aurélie Cobat, Bertrand Boisson, Qian Zhang, Stéphanie Boisson-Dupuis, Laurent Abel, Jean-Laurent Casanova

**Affiliations:** ^a^St. Giles Laboratory of Human Genetics of Infectious Diseases, Rockefeller Branch, The Rockefeller University, New York, NY 10065; ^b^Division of Pediatric Allergy and Immunology, Necmettin Erbakan University, Meram Medical Faculty, Konya 42080, Turkey; ^c^Institute of Physiological Chemistry, Technische Universität Dresden, Dresden 01307, Germany; ^d^Institute of Medical Genetics, School of Medicine, Cardiff University, Cardiff CF14 4XN, United Kingdom; ^e^Laboratory of Human Genetics of Infectious Diseases, Necker Branch, INSERM UMR1163, Paris 75015, France; ^f^Paris Cité University, Imagine Institute, Paris 75015, France; ^g^Department of Pediatrics, Necker Hospital for Sick Children, Paris 75015, France; ^h^HHMI, New York, NY 10065

**Keywords:** AG-gain, branchpoint, human genetics, intronic variant, splicing

## Abstract

Human genetic variants that introduce an AG into the intronic region between the branchpoint (BP) and the canonical splice acceptor site (ACC) of protein-coding genes can disrupt pre-mRNA splicing. Using our genome-wide BP database, we delineated the BP-ACC segments of all human introns and found extreme depletion of AG/YAG in the [BP+8, ACC-4] high-risk region. We developed AGAIN as a genome-wide computational approach to systematically and precisely pinpoint intronic AG-gain variants within the BP-ACC regions. AGAIN identified 350 AG-gain variants from the Human Gene Mutation Database, all of which alter splicing and cause disease. Among them, 74% created new acceptor sites, whereas 31% resulted in complete exon skipping. AGAIN also predicts the protein-level products resulting from these two consequences. We performed AGAIN on our exome/genomes database of patients with severe infectious diseases but without known genetic etiology and identified a private homozygous intronic AG-gain variant in the antimycobacterial gene *SPPL2A* in a patient with mycobacterial disease. AGAIN also predicts a retention of six intronic nucleotides that encode an in-frame stop codon, turning AG-gain into stop-gain. This allele was then confirmed experimentally to lead to loss of function by disrupting splicing. We further showed that AG-gain variants inside the high-risk region led to misspliced products, while those outside the region did not, by two case studies in genes STAT1 and IRF7. We finally evaluated AGAIN on our 14 paired exome-RNAseq samples and found that 82% of AG-gain variants in high-risk regions showed evidence of missplicing. AGAIN is publicly available from https://hgidsoft.rockefeller.edu/AGAIN and https://github.com/casanova-lab/AGAIN.

## Significance

The search for candidate variants underlying human disease typically focuses on coding regions and essential splice sites, mostly ignoring noncoding intronic variants. Thanks to our previously developed BPHunter, we precisely delineated the intronic segments from branchpoints to acceptor sites (BP-ACC) of all human introns, in which the AG-gain variants could interfere with constitutive splicing and result in misspliced products. Here, we developed AGAIN as a genome-wide method to systematically, efficiently, and precisely pinpoint intronic AG-gain variants in this region. AGAIN retrospectively captured reported pathogenic AG-gain variants for comprehensive analyses, and AGAIN prospectively detected new AG-gain variants that were successfully validated. AGAIN would permit the selection of promising intronic variants with biological significance, underlying rare/common and germline/somatic genetic diseases.

**Fig. 1.**
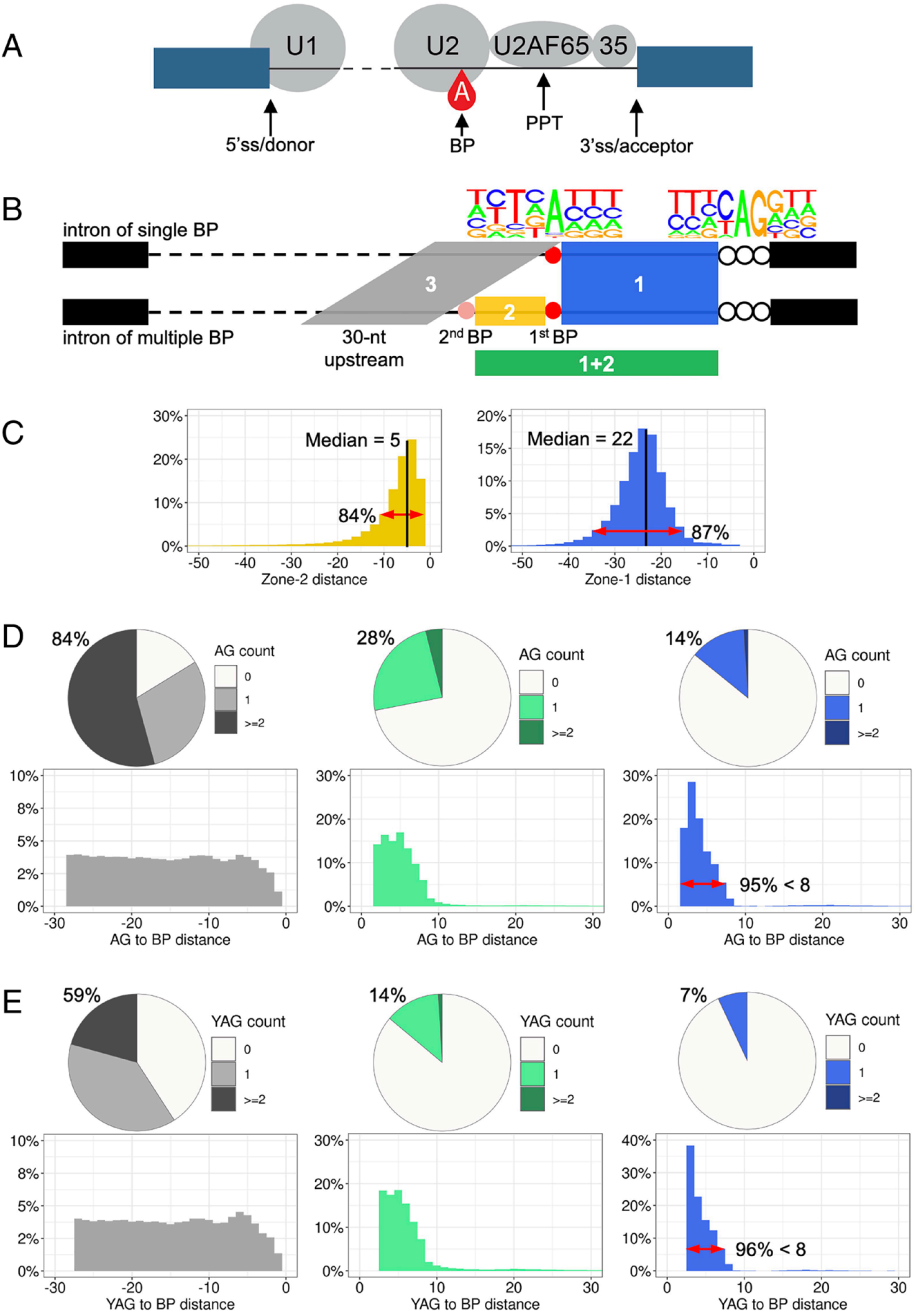
Schematics of splicing elements, zones of interest, and naturally occurring AG/YAG in the human reference genome. (*A*) Schematic of the main *cis*/*trans*-acting elements involved in splicing. (*B*) Definition of three zones of interest in this study and nucleotide frequencies around BP and ACC. (*C*) Distance distribution of zones 1 and 2. (*D* and *E*) Naturally occurring AG/YAG in the three zones: the proportion of zone sequences included AG/YAG (*Upper*) and the distance from AG/YAG to BP (*Lower*).

RNA splicing is a posttranscriptional process that transforms precursor mRNA (pre-mRNA) to mature mRNA, thereby providing a template for protein translation. Splicing is regulated by an array of *cis*-acting elements (RNA sequences with their splicing codes) and *trans*-acting elements (proteins and small nuclear RNAs (snRNAs) that bind to the *cis*-acting elements), which together constitute a complex and dynamic spliceosome (1, 2). The branchpoint (BP) is a single nucleotide generally located 20 to 40 nucleotides (nt) upstream from the canonical AG dinucleotides of the splice acceptor site (ACC, also known as the 3′ splice site or 3′ss), and the BP is one of the first elements to be recognized by the spliceosome (1, 3). Together with the recognition of ACC and the polypyrimidine tract (PPT) by U2AF35 and U2AF65, respectively, in the major spliceosome, the splicing machinery is formed at the 3′ end of the intron in pre-mRNAs (Fig. 1*A*). Splicing is a critical process for the correct generation of gene products, but its complexity renders it vulnerable to a range of deleterious variants that can perturb any part of the splicing process, yielding defective gene products.

An intronic variant disrupting BP could potentially lead to missplicing of pre-mRNA at the biochemical level (exon skipping or intron retention, completely or partially) and result in a loss-of-expression/function gene product, with pathological impact. We previously developed BPHunter software and database for the genome-wide detection of intronic BP variants (1). In the application of BPHunter to the 48 published pathogenic BP variants, eight of them were missed. Further analysis of these eight variants revealed that, rather than disrupting the BP itself, they all created AG dinucleotides between the BP and ACC. The original reports noted complete/partial exon skipping or intron retention, rather than the use of a novel acceptor site at the newly created AG (*SI Appendix*, Fig. S1). We therefore hypothesized that intronic variants introducing AG dinucleotides (AG-gain variants) into the BP-ACC region may promote premature binding with U2AF35 that aims to recognize the acceptor site, thereby sterically interfering with the recognition of PPT by U2AF65 and the formation of the spliceosome complex at the 3′ end of the intron, near the canonical splice acceptor site.

Molecular biologists and geneticists have been aware of the functional impact and deleterious role of such AG-gain variants for some time. In 1993, Smith et al. proposed a scanning and competition mechanism for AG recognition from the BP to its downstream region (4). They concluded that the proximity to the BP is a major determinant for AG recognition and competition, and the steric effect of the spliceosome renders an AG less competitive when it is <12 nt from the BP. Chua and Reed studied the distance-dependent competition of an upstream AG versus a downstream AG to be selected as the acceptor site (5). They used one specific intron as the template and found that the upstream AG is more competitive when located <6 nt away from the downstream AG. Vorechovský analyzed the variants that lead to the use of aberrant splice acceptor sites, about one-half of which created AG dinucleotides (6). However, the variant collection was based on their spliced outcome (creating new acceptor sites), not based on their nature (creating AG dinucleotides). Hence, many AG-gain variants leading to exon skipping or intron retention were missed in the analysis.

Recently, some studies have used the “AG-exclusion zone” (AGEZ) to represent the region from BP to ACC in which no AG should be found (7, 8). Wimmer et al. analyzed 91 variants of gene *NF1* in the AGEZ, of which 49 variants gained AG and 24/49 (49%) led to exon skipping (7). However, this study defined the AGEZ as the region from ACC to its next upstream AG and collated the intronic variants of interest without using any BP data. Bryen et al. screened and analyzed AG-gain variants in the AGEZ from the ClinVar and LOVD databases (8). However, this study had several limitations. First, to define AGEZ, it only used the Branchpointer dataset, an incomplete BP dataset, resulting in 27% of introns being excluded from the study. Second, it assumed a pathogenic label to denote splice-altering and a benign label to denote non-splice-altering, to separate variants into two groups for comparison; however, a benign variant could lead to missplicing biochemically, without physiological impact. Finally, it did not attempt literature review to identify experimental mRNA phenotyping data and therefore did not contain any analysis of the missplicing consequences. As the paper also mentioned, ClinVar and LOVD entries are not always rigorously reviewed and rarely provide evidence for pathogenicity.

Human genetic studies have occasionally reported intronic variants that created AG dinucleotides close to ACC, with missplicing consequences at the biochemical and physiological levels. For example, the earliest report of such a variant was in gene *HBB* from a patient with beta-thalassemia in 1981 (9), and a more recent example of such a variant was in gene *STAT1* from a patient with primary immunodeficiency (10). These pathogenic AG-gain variants were mostly identified by analyzing all variants in known disease-causing genes from small cohort studies. Therefore, some challenges remain to be addressed: i) a genome-wide and precise map of the BP-ACC segment of all human introns has not yet been established; ii) the in-depth characterization of reference genome and population variants in the BP-ACC region has not yet been made; iii) a comprehensive analysis of the known pathogenic AG-gain variants and their experimentally validated consequences is still lacking; and importantly, iv) an efficient tool to precisely pinpoint candidate intronic AG-gain variants in the BP-ACC region, from sequencing data, is not yet available. With the help of our previously developed BPHunter method and database, we aim to address these shortcomings, fill the gaps in our knowledge, and improve our capability to identify and investigate intronic variants underlying human diseases and traits.

## Results

### Definition of BP-ACC Regions and their Sequence Properties.

In BPHunter, we established a comprehensive genome-wide database of human BP, with 32% and 68% of introns having single and multiple BP sites, respectively (1). Herein, we first focused on the canonical transcripts of protein-coding genes (one transcript per gene) and extracted 176,841 first BP (the closest to ACC) and 121,027 second BP (the next closest to ACC in introns of multiple BP) in 176,841 introns from 17,488 canonical transcripts of 17,488 genes (*Methods*). We defined three zones of interest (Fig. 1*B*): (zone 1) from the first BP+1 position to the ACC-4 position, for all introns; (zone 2) from the second BP+1 position to the first BP-1 position, for introns with multiple BP; and (zone 3) 30-nt upstream from the BP-1 position (the first BP in introns of single BP, or the second BP in introns with multiple BP). We measured the sequence lengths and pyrimidine contents (Y = C/T) of these zones (Fig. 1*C* and *SI Appendix*, Fig. S2*A*). In zone 1, 87% of sequences were within a 20-nt window (15 to 35 nt), with a median length of 22 nt. Zone 1 exhibited very high pyrimidine content, with a median percentage of pyrimidine at 76% reflecting the PPT in this region. Zone 2 was a narrow region, as the second BP was generally located close to the first BP, with 84% of them less than 10-nt upstream away and with a median length of 5 nt. The percentage of pyrimidines in zone 2 was reduced as compared with zone 1, but still having a median of 67%. Zone 3 had a fixed window of 30 nt, as a control region of zone 1 with a comparable length. Zone 3 was characterized by evenly distributed pyrimidines and purines (A/G), as its pyrimidine content was mostly around 50%. These results suggested that the low purine content in zone 1 may be related to the depletion of AG in this region, and the genetic variants occurring in zone 1 may be well covered by whole-exome sequencing (WES) due to its closeness to the following exon, without the requirement for whole-genome sequencing (WGS) data to detect them.

### Naturally Occurring AG Dinucleotides in the Human Reference Genome.

We searched for naturally occurring AG dinucleotides within these zones and found that 14% of zone 1 sequences, 28% of zone 1+2 sequences, and 85% of zone 3 sequences included at least one AG in the human reference genome (Fig. 1*D*). Measuring the distances from those naturally occurring AG to BP in zone 1, 95% of these were located within 7-nt downstream from BP. It showed that AG is highly depleted from BP+8 onward, with only 0.7% [=0.14*(1 − 0.95)] of introns having AG in this range. With a narrow extension (5-nt median length) from zone 1 to zone 1+2, the frequency of naturally occurring AG doubled from 14 to 28%. AG were found in 84% of zone 3 sequences, which is very close to the theoretical probability (87%) of finding at least one AG in a random 30-nt sequence, assuming a uniform and independent A/C/G/T distribution at each position. AG dinucleotides in zone 3 were also evenly distributed across its entire length. Furthermore, as the ACC-3 position is highly conserved as a pyrimidine (Fig. 1*B*), we also searched for the naturally occurring YAG and found that 7% of zone 1 contained YAG, with 96% of them being located within 7-nt downstream from BP. This showed that YAG is extremely depleted from BP+8 onward, with only 0.28% [=0.07*(1 − 0.96)] of introns having YAG within this range (Fig. 1*E*). In addition, we tested Smith et al.’s hypothesis that naturally occurring AG may be concealed inside RNA stem-loop structures, thereby not exposing them to other interactions (4). We observed that, of the 14% of zone 1 sequences that included AG, 44.4% of these AG could perfectly base-pair within zone 1, when we simulated the RNA structures with minimum free energy (11) (*SI Appendix*, Fig. S2 *B* and *C*). Here, we showed that naturally occurring AG/YAG are extremely depleted in the [BP+8, ACC-4] region and that about half of the naturally occurring AG could be concealed within RNA structures.

### AGAIN: Genome-wide Detection of Intronic AG-gain Variants in the BP-ACC Region.

**Fig. 2.**
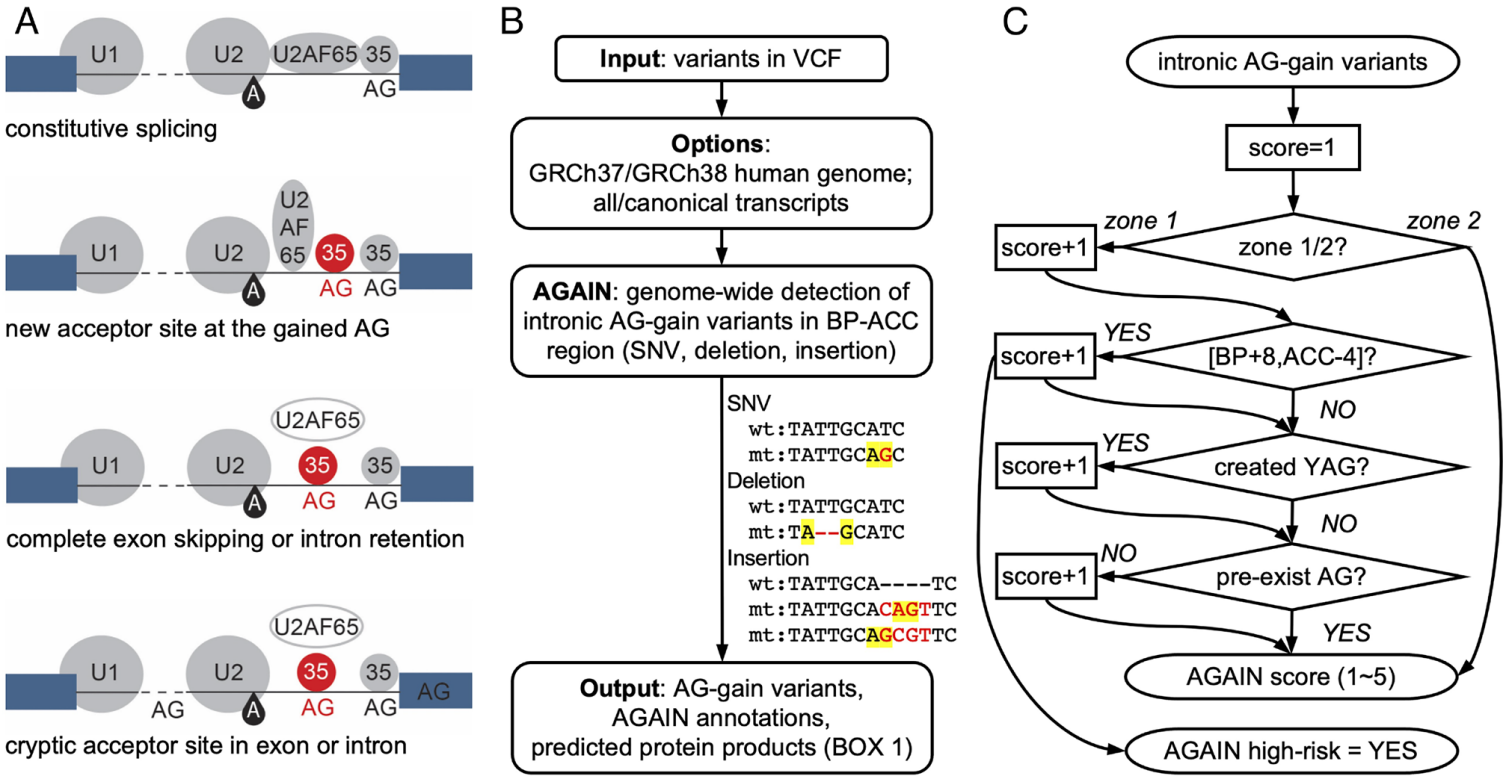
Detection of intronic AG-gain variants. (*A*) Schematics of the disrupted splicing mechanism by AG-gain variants and their consequences. (*B*) Schematic workflow of AGAIN software and types of AG-gain variants with artificial examples. (*C*) Scoring scheme in computing the AGAIN score.

Given the eight pathogenic AG-gain variants that displayed missplicing in our BPHunter study (1), and the strong evidence for AG/YAG depletion after 8-nt downstream from BP, we hypothesized that the newly created AG dinucleotides in the BP-ACC region, especially in the [BP+8, ACC-4] region, may interfere with the recognition of PPT and disrupt the formation of the spliceosome complex at the 3′ end of the intron. We therefore developed the AGAIN software for the genome-wide detection of intronic AG-gain variants located between BP and ACC, which are highly likely to lead to aberrant mRNA splicing and the formation of defective gene products (Fig. 2*A*). AGAIN takes variants in VCF format as input and outputs single-nucleotide variants (SNVs) and indels (micro-insertions and -deletions) that accidentally introduced a new AG (Fig. 2*B*), with informative annotations (Box 1). AGAIN also provides a high-risk tag (yes/no for variants in the [BP+8, ACC-4] region) and a score (ranging from 1 to 5, summed up several important factors) for prioritizing candidate variants (Fig. 2*C*). Inspired by our previously developed tool, SeqTailor, that extracts DNA sequences and generates protein sequences for genetic variants (12), we enabled AGAIN to predict the protein-level outcomes from the two main missplicing consequences of AG-gain variants: a new acceptor site at the newly created AG and complete skipping of the next exon (more details in the next section). We built an algorithm for protein sequence generation: It first determines whether an AG-gain variant resides before or after the translational start codon (e.g., a start codon could appear in exon-2), checks for the reading frame, assembles the consequent coding sequence, translates into protein sequence, and generates HGVS nomenclature (13) (*SI Appendix*, Fig. S3). AGAIN was developed into a one-line command that can be implemented in large-scale analysis, and also developed as a webserver with a user-friendly interface for small-scale analysis. AGAIN serves as a practical and useful tool for selecting promising candidate intronic variants that may have biochemical and pathological impact.

Box 1. Input and output of AGAIN software

AGAIN takes variants in VCF format as input, with its first five columns as mandatory fields (CHROM, POS, ID, REF, and ALT). It provides an option for the human reference genome GRCh37/hg19 or GRCh38/hg38, and an option for all transcripts [based on the GENCODE database (14)] or canonical transcripts [based on the MANE database (15)]. It outputs SNVs and indels that introduce AG in the BP-ACC region (Fig. 2*B*), with the following annotations:

• VAR_TYPE: type of variant (snv, x nt-deletion, x nt-insertion)
• GENE: gene symbol
• TRANSCRIPT_IVS: transcript ID and intron number (e.g., ENST123456789_IVS10)
• CANONICAL: canonical transcript ID and intron number; otherwise noted as “.”
• AGAIN_ZONE: ZONE 1/ZONE 2
• AGAIN_YAG: if the AG-gain variant also fits YAG (YES/NO)
• AGAIN_BP_DIST: distance from the created AG to BP
• AGAIN_ACC_DIST: distance from the created AG to ACC
• AGAIN_HIGHRISK: if the AG-gain variant falls inside the [BP+8, ACC-4] region (YES/NO)
• AGAIN_SCORE: a score ranging from 1 to 5
• PROT_SEQ_WT: wild-type protein sequence
• PROT_SEQ_NEW_ACC: consequent protein sequence if new acceptor site is created
• HGVS_NEW_ACC: protein-level HGVS annotation if new acceptor site is created
• PROT_SEQ_EXON_SKIP: consequent protein sequence if exon skipping occurs
• HGVS_EXON_SKIP: protein-level HGVS annotation if exon skipping occurs

### Published Pathogenic AG-gain Variants.

**Fig. 3.**
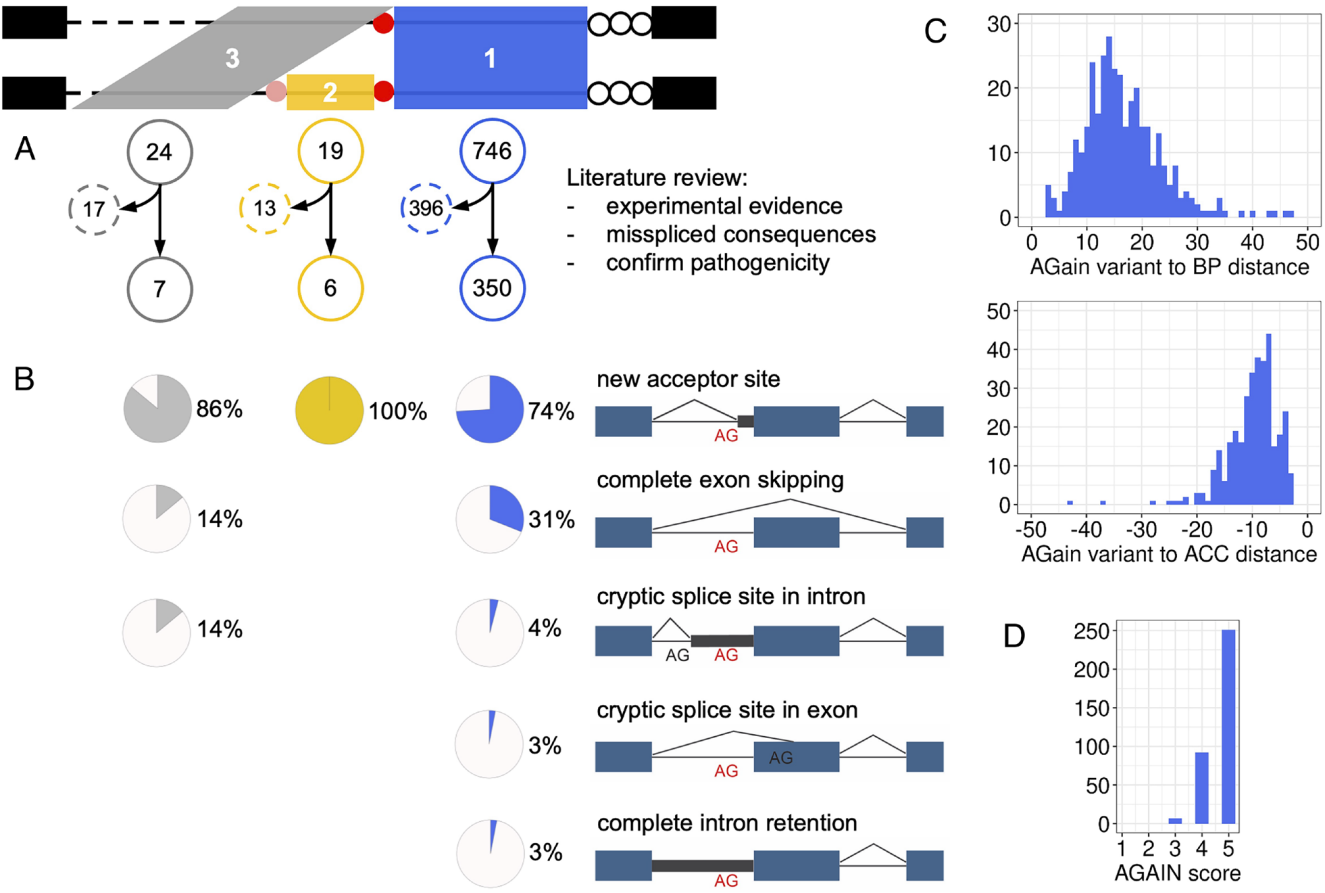
Analysis of published pathogenic AG-gain variants. (*A*) Search of the HGMD database for pathogenic AG-gain variants in three zones and literature review of each associated paper to obtain experimental missplicing evidence. (*B*) Misspliced outcomes from the reported pathogenic AG-gain variants. (*C*) The distance distribution from pathogenic AG-gain variants to BP (*Left*) and to ACC (*Right*) in zone 1. (*D*) The distribution of AGAIN scores of pathogenic AG-gain variants.

We employed AGAIN to screen all the pathogenic variants documented and curated by the Human Gene Mutation Database (HGMD) database professional version 2023.1 (16) and identified a total of 789 AG-gain variants from the three zones of interest (746, 19, and 24 variants in zones 1, 2, and 3, respectively). We further performed an in-depth literature review of each associated paper and checked each variant for its experimental validation and missplicing consequences. In so doing, we refined this collection to 350 variants in zone 1 (342 SNVs, 5 micro-insertions and 3 micro-deletions), 6 variants in zone 2 (all SNVs), and 7 variants in zone 3 (all SNVs), all of which had been experimentally validated at the biochemical level (by exon trapping, RNA-seq, western blot, etc.) and their gene-disease associations were confirmed at the clinical level (by literature search, OMIM, etc.) (Fig. 3*A* and Dataset S1). The earliest pathogenic intronic AG-gain variants, as we found, was published in 1981, with the report of an intronic base substitution in gene *HBB* from a Greek girl with beta-thalassemia (9). Focusing on the 350 AG-gain variants in zone 1, their mode of inheritance included 130 (37%) autosomal dominant, 116 (33%) autosomal recessive, 39 (11%) autosomal dominant and recessive, 17 (5%) X-linked dominant, 28 (8%) X-linked recessive, and 20 (6%) unreported (*SI Appendix*, Fig. S4*A*). Their biochemical deleteriousness and clinical pathogenicity were caused by several missplicing consequences (one variant could have more than one consequence): 258 (74%) created a new acceptor site at the newly introduced AG, leading to the insertion of intronic nucleotides into coding sequence (66 in-frame and 192 out-of-frame); 110 (31%) resulted in the complete skipping of the next exon; 14 (4%) and 10 (3%) activated the cryptic splice site in the intron and exon, respectively, and 11 (3%) showed complete intron retention (Fig. 3*B* and *SI Appendix*, Fig. S4*B*). The creation of YAG was associated with the creation of new acceptor sites. Of the 258 variants that created new splice sites, 229 (89%) exhibited YAG; of the 92 variants that did not create new splice sites, 72 (78%) exhibited YAG, which is markedly lower (*P*-value = 0.0122 by Fisher’s exact test). We measured the distance from AG-gain variants to BP and ACC, respectively, in which 330/350 (94%) were ≥8 nt from BP, and 334/350 (95%) were ≤17 nt from ACC (Fig. 3*C*). No noticeably distinct pattern was observed when we separately focused on the variants that created new acceptor sites or skipped exons (*SI Appendix*, Fig. S4 *C* and *D*). Based on these analyses and the distribution of AGAIN scores (Fig. 3*D*), we recommend the prioritization of AG-gain variants in the high-risk [BP+8, ACC-4] region with AGAIN score ≥3 as the promising candidates. This analysis also yielded an estimated minimum prevalence for pathogenic intronic AG-gain variants among all variants causing inherited diseases, which is 0.1 to 0.2% based on HGMD. This may however be regarded as a conservative estimate. With the application of AGAIN, we expect an increased number of pathogenic intronic AG-gain variants that would be rapidly identified and timely validated.

### Evaluation of SpliceAI on the Published Pathogenic AG-gain Variants.

**Fig. 4.**
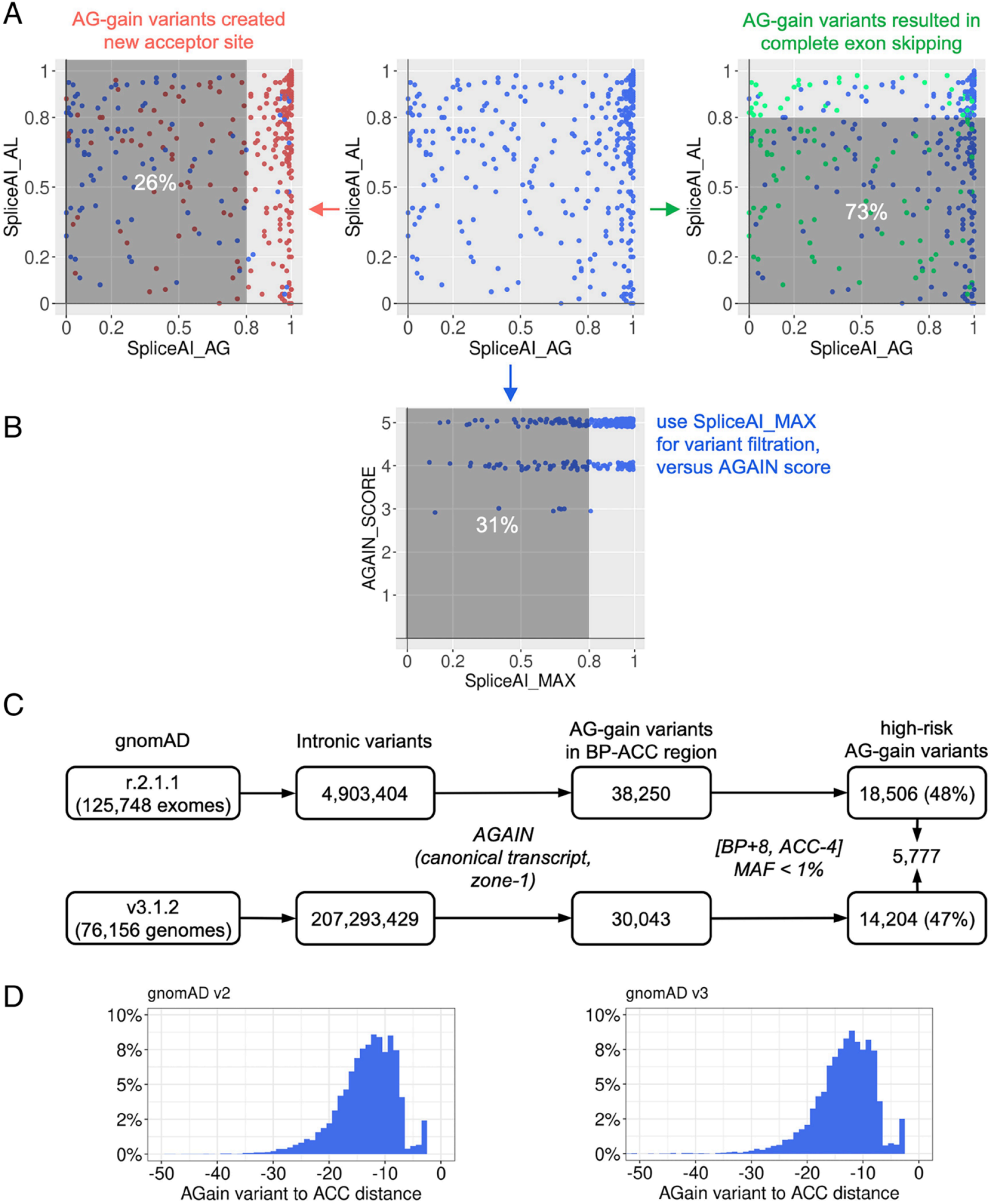
Pathogenic AG-gain variants scored by SpliceAI and population AG-gain variants in gnomAD. (*A*) Evaluation of published pathogenic AG-gain variants by computing their SpliceAI scores for acceptor gains and losses. The variants that created new acceptor sites are highlighted in red, and the variants that resulted in complete exon skipping are highlighted in green. (*B*) AGAIN score versus the maximum SpliceAI score (acceptor gain or loss). (*C*) Search for intronic AG-gain variants in the human population. (*D*) Location of AG-gain variants to the canonical acceptor sites, in gnomAD v2 and v3.

We tested SpliceAI (17) on these published pathogenic AG-gain variants that were identified from the HGMD by AGAIN. SpliceAI computes probability scores for variants that are predicted to gain or loss of acceptor sites (DS_AG, DS_AL), which exactly correspond to the creation of new acceptor sites and exon skipping, respectively. SpliceAI suggested a cutoff of 0.8 for the high-precision predicted likelihood of altered splicing. By performing SpliceAI on the 350 pathogenic AG-gain variants in zone 1 with documented missplicing consequences (Fig. 4*A*), we found that 26% of the 258 variants that created new acceptor sites had their DS_AG scores below the cutoff, while 73% of the 110 variants that gave rise to exon skipping had their DS_AL scores below the cutoff. We then used the maximum of SpliceAI score (the higher one of DS_AG and DS_AL) and found that 31% of all 350 pathogenic AG-gain variants were still below the cutoff (Fig. 4*B*). We acknowledge the usefulness of SpliceAI in genome-wide screening of variants with missplicing potential. Our analysis found that SpliceAI works reasonably well on AG-gain variants for acceptor-site gains (with 26% missed), but might be underpowered to predict acceptor-site losses by AG-gain variants (with 73% missed). This analysis provides support for the use of not only the machine-learning trained scores (e.g., SpliceAI), but also the biological mechanism-inspired methods, such as AGAIN and BPHunter (1). We aim to dissect the specific mechanism through which each individual variant may interfere with constitutive splicing and to establish a more precise and informative interpretation of genetic variants of potential biological implications and pathological significance.

### AG-gain Variants in the Human Population.

With our current understanding of the deleterious potential of intronic AG-gain variants in the BP-ACC region, we extracted intronic variants of human protein-coding genes from the gnomAD database [r.2.1.1 (v2) of 125,748 WES data and v3.1.2 (v3) of 76,156 WGS data, respectively] (18). By performing AGAIN and focusing on canonical transcripts of protein-coding genes, we identified 38,250 and 30,043 AG-gain variants in zone 1 from gnomAD v2 and v3, respectively, of which 18,506 (48%) and 14,204 (47%) (7,555 overlapping) were in the high-risk [BP+8, ACC-4] region with minor allele frequencies (MAF) <1% (Fig. 4*C* and Datasets S2 and S3). The distances between these AG-gain variants and their acceptor sites were distributed similarly in v2 and v3 (Fig. 4*D*), suggesting a good detection coverage of AG-gain variants from WES. These population AG-gain variants in gnomAD v2 and v3 comprised 57.5% and 57.9% singleton variants [allele count (AC) = 1], 42.3% and 41.6% rare variants (MAF < 1%), and 0.2% and 0.5% common variants (MAF ≥ 1%), respectively. We also identified the predicted loss-of-function (pLOF) variants (stop-gain, frameshift, and essential splice site variants) from gnomAD, and the high-risk AG-gain variants is ~5 to 6% of the pLOF category. A further comparison between six groups of intronic variant in the human population (AG-gain variants, BP variants, essential acceptor site variants, all other variants within the BP-ACC region, and one million random intronic variants) revealed that AG-gain and BP variants have lower frequencies than the other intronic variants in the BP-ACC region and the intronic background, but higher frequencies than the essential acceptor splice site variants (*SI Appendix*, Fig. S5 *A* and *B*). Moreover, the population pLOF variants are particularly useful for selecting new candidate genes/variants. We therefore upgraded our PopViz webserver (19) to include the high-risk AG-gain and BP variants in its rapid integrative visualization of population variants and their deleteriousness scores. PopViz has been widely applied to reinforce or reject the plausibility of candidate disease-causing genes and to prioritize the candidate variants to be tested (19).

### A Homozygous Germline Intronic AG-gain Variant of *SPPL2A* in a Patient with Mycobacterial Disease.

**Fig. 5.**
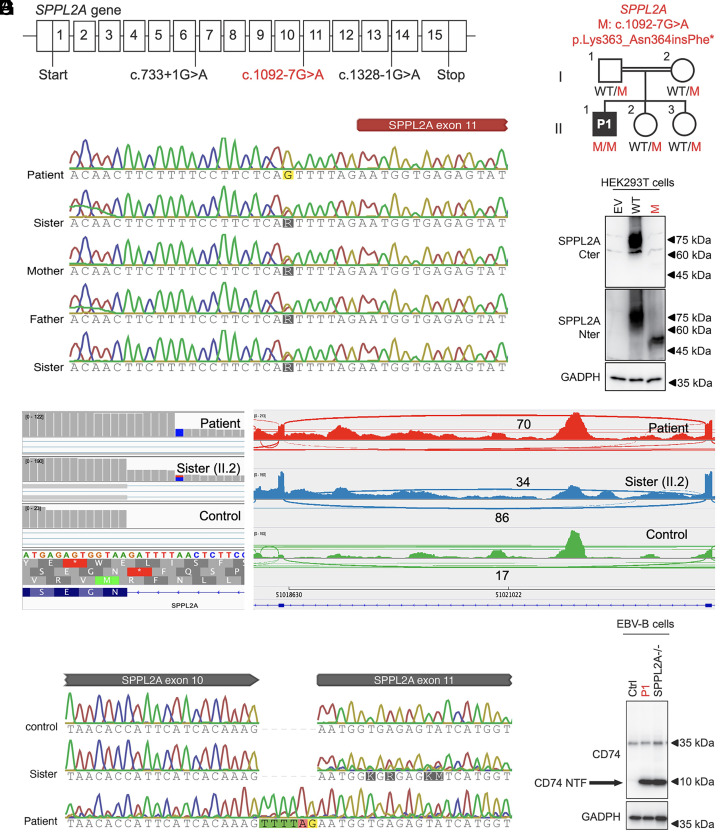
Identification and validation of a homozygous intronic AG-gain variant of *SPPL2A* in a patient with mycobacterial disease. (*A*) Gene structure of *SSPL2A* and its reported pathogenic variants (black) and the intronic AG-gain variant (red). (*B*) Pedigree of the patient’s family, in which the patient is the only homozygous carrier and the other family members are heterozygous carriers. (*C*) Sanger sequencing of genomic DNA of family members. (*D*) RNA-seq alignment and splice-junction reads in the Sashimi plot. (*E*) Sanger sequencing of the RT-PCR products from mRNA. (*F*) Immunoblotting analysis of the variant in HEK293T cells. (*G*) Immunoblotting analysis of CD74 N-terminal fragments in EBV-B cells.

We performed AGAIN to screen our WES/WGS database of ~20,000 patients with severe infectious diseases (viral, bacterial, and fungal), whom have no known genetic etiology yet. We focused our analysis on 345 known inborn error of immunity (IEI) genes (20) and extracted homozygous variants with AGAIN’s high-risk tag. We then focused on a homozygous germline intronic AG-gain variant (chr15-51018581-T-C, c.1092-7G>A) of a known antimycobacterial gene *SPPL2A* in a patient with mycobacterial disease (Fig. 5*A*). *SPPL2A* encodes SPPL2a, an intramembrane protease that degrades CD74 (HLA invariant chain) in antigen-presenting cells (21, 22). Human SPPL2a deficiency causes mycobacterial disease by decreasing the number of conventional type 2 dendritic cells (cDC2) and impairing IFN-γ production by helper T cells (21). This AG-gain variant introduced a new AG dinucleotide 6-nt upstream from the ACC of intron-10 and 18-nt downstream from the BP, with an AGAIN score 5 and a MAF~1e-6 in gnomAD. Sanger sequencing confirmed homozygosity of the variant in the patient, whereas all other heterozygous family members were healthy (both parents and two sisters) (Fig. 5 *B* and *C*). AGAIN also predicted the resulting protein-level products from the two major missplicing outcomes: If a new acceptor site was created, it would result in an in-frame insertion of six nucleotides that includes a stop codon (p.Lys363_Asn364insPhe*); if complete exon skipping were to occur, it would give rise to an in-frame deletion of 21 amino acids (p.Asn364_Lys382del). We therefore hypothesized that this homozygous intronic AG-gain variant was responsible for the aberrant splicing of *SPPL2A* transcripts and the production of a loss-of-function SPPL2a protein, leading to autosomal recessive (AR) SPPL2a deficiency in the patient.

### The Homozygous Intronic AG-gain Variant of *SPPL2A* Is Loss of Function.

We set out to validate the homozygous intronic AG-gain variant of *SPPL2A*. Adopting an unbiased approach, we first performed RNA-seq on the mRNA from peripheral blood mononuclear cells (PBMCs) of the patient, his healthy sister (heterozygous carrier, II.2), and an unrelated control (Fig. 5*D*). We observed that in the patient’s RNA-seq data, all 70 splice-junction reads were aligned to the predicted new acceptor site that is 6-nt shifted into the intron, including an in-frame stop codon (p.Lys363_Asn364insPhe*), without the use of the canonical acceptor site, and without any detectable exon skipping. The unrelated control had all 17 splice-junction reads located at the canonical acceptor site, whereas the patient’s heterozygous sister exhibited a ratio of 86/34 splice-junction reads at the canonical/new acceptor sites (Fig. 5*D*). This 6-nt insertion was also confirmed by Sanger sequencing of the RT-PCR products from the patient’s mRNA (Fig. 5*E*). The patient had comparable levels of *SPPL2A* mRNA expression, as determined by RT-qPCR, thereby allowing us to exclude the action of nonsense-mediated decay (NMD) (*SI Appendix*, Fig. S6). We next hypothesized that the truncated SPPL2a protein (p.Lys363_Asn364insPhe*) is a loss of function. First, no protein was detected by immunoblotting analysis of HEK293T cells transfected with cDNA corresponding to the 6-nt insertion with a C-terminal SPPL2A antibody, whereas a less abundant and truncated protein can be detected with an N-terminal SPPL2a antibody (Fig. 5*F*). Second, we tested the accumulation of CD74 N-terminal fragments, a hallmark of cells with complete AR SPPL2a deficiency (21), in the patient’s EBV-B cells. As expected, immunoblotting showed a strong accumulation of CD74 N-terminal fragments in the patient's cells, similar to a reported patient with known AR SPPL2a deficiency (Fig. 5*G*). Therefore, AGAIN identified a novel form of complete AR SPPL2a deficiency in a patient with mycobacterial disease, thereby neatly illustrating the potential of intronic AG-gain variants in the etiology of human genetic disease.

### Splicing Impact of Two Intronic AG-gain Variants of *STAT1* Inside and Outside High-Risk Regions.

**Fig. 6.**
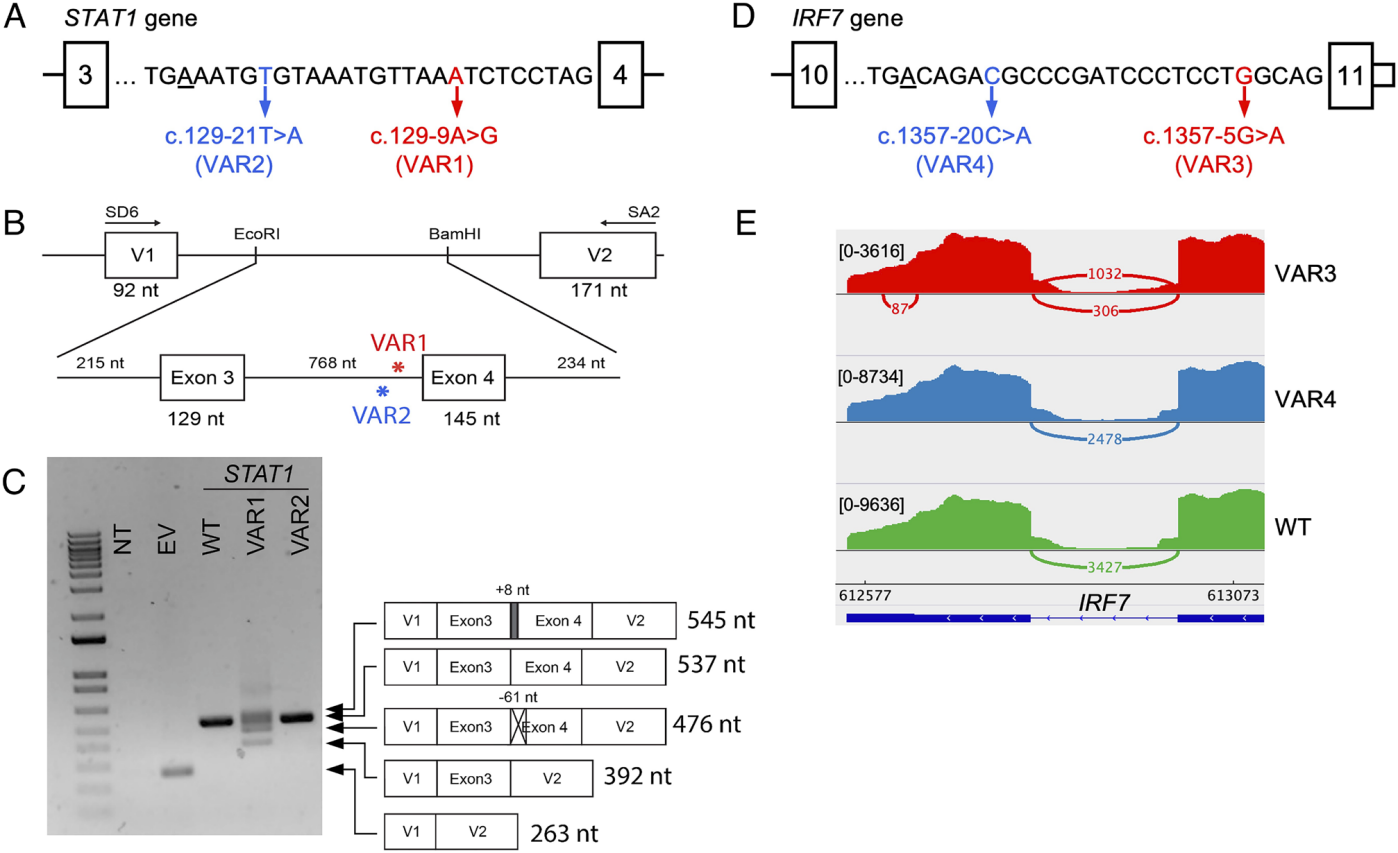
Impact on splicing by intronic AG-gain variants from the same intron of *STAT1* and *IRF7* that are inside and outside the high-risk region. (*A*) Two intronic AG-gain variants of *STAT1* that are inside (VAR1) and outside (VAR2) the high-risk region, where BP is underscored. (*B*) Schematic diagram of the exon trapping experiment. (*C*) Agarose gel electrophoresis of RT-PCR products using primers within pSPL3 exon V1 and exon V2 from nontransfected (NT); transfected COS7 cells with empty vector (EV); and pSPL3 vectors carrying WT, VAR1, and VAR2 of *STAT1*. WT and mutant transcripts, determined by PCR amplicon sequencing, are depicted to the right side of the gel image. (*D*) Two intronic AG-gain variants of *IRF7* that are inside (VAR3) and outside (VAR4) the high-risk region. (*E*) Sashimi plot of RNAseq reads mapped to exon 10, exon 11, and the splice junction of *IRF7*, from VAR3, VAR4, and WT samples.

We defined AG-gain variants within the [BP+8, ACC-4] region as high-risk, due to the extreme depletion of AG in this segment of the human reference genome. We used AGAIN to screen our in-house WES/WGS database of patients with severe infectious diseases and applied the following selection criteria: i) focusing on 345 known IEI genes, ii) requiring at least one AG-gain variant inside a high-risk region and another outside such region (i.e., [BP+1, BP+7]) in the same intron of the same gene, and iii) confirming the variants of good sequencing and calling quality. We thereby identified two such AG-gain variants in intron-3 of gene *STAT1* (Fig. 6*A*): one variant inside the high-risk region (VAR1: chr2-191873842-T-C, c.129-9A>G, MAF = 0 in gnomAD), which creates an AG 16-nt downstream from BP, in a patient (heterozygous) with severe COVID-19 infection; the other variant outside the same high-risk region (VAR2: chr2-191873854-A-T, c.129-21T>A, MAF~1e-4 in gnomAD), which introduces an AG 5-nt downstream from BP, in 39 individuals (all are heterozygous) with a mixture of 13 different clinical phenotypes. *STAT1* is a signal transducer and transcription activator that mediates immune responses to interferons in viral infections, and its deleterious variants have been reported underlying life-threatening COVID-19 pneumonia (23). We then conducted exon trapping to test the splicing outcomes from these two variants (Fig. 6*B*). We found that VAR2 encoded transcripts that were all like the wild type (WT), while VAR1 encoded several spliced products: 40% of transcripts lacked the first 61 nt of exon-4, 30% of transcripts completely lacked exon-4, 28% of transcripts used the newly created AG that retained 8 nt of intron-3, and only 2% of transcripts were identical to WT (Fig. 6*C*).

### Splicing Impact of Two Intronic AG-gain Variants of *IRF7* Inside and Outside High-risk Regions.

From the previous searching results, we identified another two such AG-gain variants in intron-10 (last intron) of gene *IRF7* (Fig. 6*D*): one heterozygous variant inside the high-risk region (VAR3: chr11-612805-C-T, c.1357-5G>A, MAF = 0 in gnomAD), which creates an AG 21-nt downstream from BP, in a patient with severe COVID-19 infection; the other heterozygous variant outside the high-risk region (VAR4: chr11-612820-G-T, c.1357-20C>A, MAF = 0 in gnomAD), which introduces an AG 6-nt downstream from BP, in a COVID-19 control individual (asymptomatic after COVID-19 infection). *IRF7* encodes interferon regulatory factor 7, which is a key transcriptional regulator of type I interferon-dependent immune responses, and its deleterious variants have also been reported underlying life-threatening COVID-19 pneumonia (23). Due to the technical difficulty in designing exon-trapping for the last exon and 3′UTR region, we performed RNA-seq for three samples (VAR3, VAR4, and WT) to examine their splicing outcomes (Fig. 6*E*). We obtained a similar number of total reads from the three samples (VAR3: 35.8M, VAR4: 36.2M, and WT: 36.3M, respectively), but the number of reads aligned to *IRF7* was significantly lower for VAR3 (7.8K, 19.4K, and 22.5K, respectively). VAR3 sample had only 35% of *IRF7* reads of WT and 40% of *IRF7* reads of VAR4, suggesting that the VAR3 may have disrupted the spliceosome in recognizing the splice sites and the RNA polymerase elongation, which largely reduced the gene expression efficiency. We further checked the splice-junction reads between exon 10 and exon 11 and found VAR4 and WT had all their splice-junction reads in use of the canonical ACC (2,478 and 3,427, respectively), as expected. However, VAR3 had 1,032 splice-junction reads in use of the canonical ACC, while 306 (~30%) splice-junction reads in use of the AG-gain site (Fig. 6*E*). This could be because VAR3 created a trinucleotide TAG that is less preferred than CAG to be an acceptor site. Based on these two case studies, we recommend that AG-gain variants in the high-risk [BP+8, ACC-4] regions should be prioritized for functional validation, and AGAIN has the capability to clearly tag them based on the intron-specific distribution of branchpoints.

### Detection of Intronic AG-gain Variants and Validation of their Consequences from Paired WES and RNA-seq Data.

We searched for genome-wide AG-gain variants and tested their impact on splicing at the biochemical level, by revisiting 14 individuals’ RNA-seq data from our previously published studies (24–28), for which we had paired WES-RNAseq data available in-house. We performed AGAIN to screen these individuals’ WES data (in any protein-coding gene, as we aimed to exclusively focus on the evidence on missplicing, not on pathogenicity) and identified 23 intronic AG-gain variants in the high-risk [BP+8, ACC-4] region with AGAIN scores ≥3. After examining their WES read alignment, 3 variants were removed due to failed quality control, leaving 20 variants to be validated. We then checked their corresponding RNA-seq data and found that 9 variants were in genes with no or very low RNA-seq expression, which were discarded for further analysis. Of the remaining 11 AG-gain variants that had sufficient RNA-seq read coverage, we found that 9 (82%) of them yielded evidence of missplicing (new acceptor site, exon skipping, and intronic retention) (*SI Appendix*, Fig. S7). A new acceptor site was observed in 5 cases, intronic retention in 3 cases, and exon skipping in 2 cases (one variant could have more than one consequence). It is also important to mention that the RNA-seq data used for this biochemical-level validation of AG-gain variants were originally generated in different studies with different configurations (experimental protocols, cell types, read lengths, read depths, and sequencing machines), and NMD inhibitors were not used to retain misspliced transcripts prior to RNA-sequencing. This analysis only estimated an approximate proportion of AG-gain variants in high-risk region that may give rise to missplicing, due to the heterogeneous nature of the data.

## Discussion

In the search for candidate variants in protein-coding genes from patients, current methods mostly focus on nonsynonymous variants in coding regions and variants at essential splice sites, while neglecting to consider the vast number of noncoding intronic variants. Where a study plans to include intronic candidate variants in the investigation, analytical approaches may include i) checking through all the detected intronic variants in known disease-causing genes and manually examining their genomic locations and nucleotide changes to infer their potential functional impact; this would be a daunting task for unknown disease genes, unless preselected by other approaches, such as linkage; ii) computing and ranking the intronic variants by deleteriousness, evolutionary conservation, and missplicing scores (17, 29); iii) testing for the enrichment of specific intronic variants in a case–control study or in a linkage analysis; iv) using the human population MAF to remove common intronic variants and to prioritize private/rare intronic variants (18, 19); v) applying BPHunter software to search for intronic variants that may disrupt BP and hence splicing (1); and vi) clustering genetic heterogeneity (pLOF variants + private/rare intronic variants with high deleterious scores + BP variants) based on the biological interactions among human genes (30).

Among the myriad intronic variants (for example, 30% of all variants in gnomAD v2 of WES data are intronic, and 81% of all variants in gnomAD v3 of WGS data are intronic), AG-gain variants in high-risk region should be among the top-ranked intronic candidates to be considered in human genetic studies, as their functional impact has been amply documented by molecular genetic studies (4–8). Therefore, a tool permitting the systematic, efficient, and precise detection of such candidate variants has been long-waited. The application of AGAIN could be used to support the disease candidacy of a homozygous intronic variant of a known disease-causing gene, or a compound heterozygous hit with another pLOF variant (stop-gain, frameshift, essential splice site) of the same gene or a digenic/polygenic hypothesis that could suggest biochemical contributions from multiple genetic defects to the clinical phenotype. Besides the potential deleteriousness of intronic AG-gain variants, there are five other important facts to consider, which encourage a wide use of AGAIN for the selection of candidate intronic variants.

First, AG-gain variants are located close to the splice acceptor site, a region that is well covered by WES, without the necessity for WGS (Fig. 4*D*). Existing WES/WGS data can be quickly and easily rescreened by AGAIN to probe additional promising hits in known disease-associated genes, especially for patients without a genetic diagnosis. Second, indels are often ignored in analyses, and their mutated sequences are not routinely checked. However, indels can introduce a new AG dinucleotide into the BP-ACC region (Fig. 2*B*): a deletion may join an A and a G that were originally in two nonadjacent positions; an insertion may harbor an AG within its inserted sequence; or an insertion may insert a G/A next to a preexisting A/G. AGAIN is able to identify such indels, and bring them to our attention. Third, regardless of whether the AG-gain variants create new acceptor sites or lead to exon skipping, theoretically 2/3 cases will result in a frameshift within the coding sequence, which is an immediate output from AGAIN. Meanwhile, AGAIN also captures scenarios of gross transcript ablation (e.g., skipping the exon that harbors the start codon, *SI Appendix*, Fig. S3). Fourth, when an AG-gain variant creates a new acceptor site, the newly inserted intronic nucleotides could harbor an in-frame stop codon resulting in a truncated gene product. AGAIN predicts the protein-level consequences to directly signal such AG-gain = stop-gain cases. Fifth, the occurrence of a stop codon within the inserted intronic sequence is not unusual at all. The last three nucleotides of an intron are mostly YAG (64% are CAG, and 29% are TAG), whereas TAG corresponds to a stop codon. This genomic coincidence suggests that when an AG-gain variant creates a new acceptor site, there is a ~10% (29%/3 open-reading frames) probability of delivering a stop-gain impact.

We are also aware of two limitations of this work, namely that i) the experimental validation of splicing outcome from AG-gain variants inside and outside the high-risk intronic region is based on experiments performed on two genes, *STAT1* and *IRF7*, and that ii) the paired WES-RNAseq study of AG-gain variants and their missplicing consequences is based on RNAseq data that were originally generated from different studies with different configurations. These limitations suggest future directions of research: i) perform a well-designed and high-throughput experiment to generate a large-scale and high-quality dataset to systematically investigate and validate AG-gain variants and their functional impacts on splicing and ii) collect a large number of public reference RNAseq data to analyze AG-gain variants (31). These future studies may reveal new insights and knowledge: the tissue/cell type-specific BP usage and thus the definition of context-specific BP-ACC region; the sequence/distance-determinants of AG-gain variants leading to new acceptor sites or complete exon skipping; the impact of other preexisting *cis*-acting elements (intronic splicing enhancers and silencers) on splicing, etc. All of which will further improve our capability to interpret the functional impacts from intronic variants.

AGAIN promises to facilitate a variety of different types of investigations in human genetics. First, it enhances the search for promising rare germline intronic variants to be tested for biochemical consequences and physiological impact. From the HGMD database of germline variants responsible for human inherited disease, AGAIN retrospectively detected 350 reported pathogenic intronic AG-gain variants with validations, which could have been readily identified had AGAIN been available earlier. Based on HGMD, 0.1–0.2% of all pathogenic variants are intronic AG-gain variants, without using AGAIN. It is not unreasonable to expect that many more pathogenic AG-gain variants will be reported in the near future. Second, it could support the analysis of genome-wide association studies (GWASs), which have been widely applied to identify relatively common and statistically significant variants associated with human diseases and traits (32). Most GWAS variants reside in noncoding regions, without clear biological interpretation (33). The use of AGAIN and BPHunter may help to demystify the biological significance of some intronic GWAS variants that have been shown statistically significant. Third, it could assist the detection of promising somatic intronic variants, e.g., in cancers (34) and some neurological disorders (35). We screened the COSMIC database of somatic mutations (36) and identified 127 candidate high-risk AG-gain variants on genes involved in cancer initiation and progression (36, 37) (110 SNVs, 9 deletions, and 8 insertions, in 99 genes, from 124 patients with 18 cancer types) (Dataset S4). These somatic AG-gain variants may result in aberrant splicing and the synthesis of deleterious gene products, acting as cancer drivers/passengers in these patients. Therefore, the application of AGAIN should optimize the selection of promising intronic variants underlying rare or common conditions, as well as germline or somatic genetic diseases.

## Methods

### Data.

We obtained human genome sequence and gene annotation data for the GRCh37/hg19 genome assembly from the GENCODE database v44 (14) and extracted introns in genes/transcripts that were annotated as biotype = “protein-coding,” annotation tag = “basic,” and confidence level = “1 or 2.” Due to the upgrade of the GENCODE database, we realigned all BP data in BPHunter (1) to the latest collection of introns and mapped 403,952 BP to the upstream region [−3, −100] nt of the acceptor sites of 218,881 unique introns from 56,794 alternatively spliced transcripts of 17,898 protein-coding genes. Based on the canonical transcripts from the MANE database (15), and with a focus on the first/second BP mapped to introns, we retained 297,495 BP associated with 176,841 introns from 17,488 canonical transcripts of 17,488 protein-coding genes for the analysis in this paper. For the detection of intronic AG-gain variants, our AGAIN software provides an option for all or canonical transcripts. The same processes were performed for the GRCh38/hg38 genome assembly, to enable AGAIN to work with both major versions of the human genome and gene annotations.

### RNA Secondary Structure Prediction.

We used the RNAfold from ViennaRNA package (11) to predict the secondary structure of RNA sequences from BP+1 to ACC-4. RNAfold allows the intermolecular base pairing within a given RNA sequence to form a static stem-loop structure with the minimum free energy (MFE). We wrote a program to determine whether an AG is hidden inside the stem by analyzing the MFE structures.

### SpliceAI Missplicing Prediction Scores.

We used SpliceAI (17) to evaluate the intronic AG-gain variants. SpliceAI, a deep neural network model designed to predict splice junctions from RNA sequences, provides scores for the gain/loss of the acceptor/donor sites (ranging from 0 to 1, the higher the score, the higher the probability of altering the splice site). SpliceAI suggested a cutoff of 0.8 for high-precision prediction.

### Human Population Variants.

We obtained human population variants from the gnomAD database versions r.2.1.1 and v3.1.2 (18) which contain 125,748 WES and 76,156 WGS data, respectively. By focusing on protein-coding genes, we extracted 4,903,404 and 207,293,429 intronic variants from r.2.1.1 and v3.1.2, respectively. We categorized variants into singleton (AC = 1), rare (MAF < 1%), and common (MAF ≥ 1%).

### Case Report of the Patient (P1) with Mycobacterial Disease.

P1 is a 10-year-old boy, born to consanguineous Turkish parents (first cousin) in 2012. The patient had received all childhood vaccinations including BCG. His weight (16 kg; 10th percentile) and height (104 cm; 10th percentile) were low for his age. He had suffered from recurrent fever, bronchitis, and diarrhea, requiring hospitalization starting early in life that eventually resolved at two years of age. Thereafter, he started experiencing skin abscesses and lymphadenitis, with *S. aureus* identified twice. He started on intravenous immunoglobulin (IVIg) replacement therapy, which resulted in the resolution of the skin abscesses. At six and a half years of age, several occurrences of mobile lymphadenopathy (largest: 22 × 15 mm) were detected in the right axillary area and multiple ones in the cervical lymph node chain (largest: 19 × 5 mm) by ultrasonography, even though the patient was under IVIg and antibiotic prophylaxis. The size of the lymph nodes did not regress with antibiotic therapy. PPD was positive (17 mm), and the Quantiferon test was also positive. Radiologically, “tree-in-bud” branching opacities were detected by thoracic computerized tomography. Pathological examinations of the lymph nodes revealed granulomatous inflammatory lesions with central necrosis in several lymph nodes (largest: 26 × 16 mm). With a diagnosis of pulmonary TB, the patient started receiving combined anti-TB therapy including rifampicin, isoniazid, pyrazinamide, and ethambutol in 2019. The patient could not use anti-TB treatment regularly for 9 mo, and therefore, the treatment was extended to 2 y. In the follow-up, the patient had recurrent lymphadenitis and skin infection (three to four times). The patient benefited from antibiotic treatment and is still receiving IVIg. Written informed consent was obtained in the country of residence of each patient, in accordance with local regulations and with institutional review board (IRB) approval. Data analysis was conducted under the approval of the IRB of the Rockefeller University Institutional Review Board in New York, USA.

### RNA Sequencing.

Total mRNA was extracted from the cryopreserved PBMCs from the patient (homozygous carrier), her sister (heterozygous carrier), and an unrelated donor (not a carrier) using the RNeasy Plus Mini Kit (Qiagen). Full-length cDNA was generated from 1 ng total RNA with the SMART-Seq v4 Ultra Low Input RNA Kit (Clontech, 634888). One ng cDNA was then used to prepare libraries with the Nextera XT DNA Library Preparation Kit (Illumina, FC-131-1024). The libraries were sequenced on an Illumina NovaSeq 6000 sequencer with a paired-end 150 bp configuration. The raw RNA-seq fastq data were inspected to ensure high quality, and then, RNA-seq reads were mapped onto the human reference genome GRCh38 with STAR aligner (38). The alignment BAM files were then imported into IGV to visualize and examine the RNA-seq reads around the variants of interest.

### Sanger Sequencing and Real-time Quantitative PCR.

Genomic DNA was extracted from whole-blood samples from the TB patient and his family members. Nested PCR was performed to amplify the regions harboring the homozygous intronic AG-gain variant. Reverse transcription and nested PCR were performed as previously described (39) from the total mRNA extracted from PBMCs of the TB patient, his sister, and a healthy donor to amplify the regions around the 6-nt insertion resulting from the homozygous intronic AG-gain variant. Sequencing was performed as previously described (39). Real-time quantitative PCR was performed from the cDNA with two TaqMan probes targeting the exon 1–2 and 12–13 junctions of *SPPL2A* (Invitrogen; Hs01002701_g1 and Hs00607726_mH, respectively) and *GUSB* as an internal control.

### Cells and Plasmids.

The HEK293T cells (ATCC) and in-house generated EBV-immortalized B cell lines were cultured in DMEM and RPMI, respectively, supplemented with 10% FBS. A full-length WT human *SPPL2A* coding sequence (CDS) was subcloned into the pcDNA3.1(+) and pTrip-CMV-Puro-2A (a gift from Nicolas Manel, Addgene #102611) plasmids. The 6-nt insertion mutant was created by site-directed mutagenesis. Sequencing was performed to confirm the identity of the insert. Transfection into HEK293T cells, lentiviral production, and transduction into EBV-B cells were performed as previously described (39).

### Immunoblotting Analysis.

Total protein was extracted from EBV-B or HEK293-T cells in a lysis buffer containing 1% NP-40, 20 mM Tris-HCl, pH 7.4, 140 mM NaCl, 2 mM EDTA, and 50 nM NaF supplemented with Protease Inhibitor Cocktail (Roche). Protein extracts were separated by SDS-PAGE, and the resulting bands were electroblotted onto polyvinylidene difluoride membranes. The blots were incubated for 1 h with a blocking solution comprising Tris-buffered saline (TBS) with 5% Bovine Serum Albumin (BSA) (Omnipur). The following primary antibodies were diluted 1:1,000 in a washing buffer consisting of TBS plus 0.01% Tween 20 with 1% BSA and incubated overnight with the blots: rabbit anti-human SPPL2A N- and C- terminal (22), rabbit anti-human CD74 (Abcam, ab97479), and anti-GAPDH HRP (Santa Cruz Biotechnology). The blots were washed four times, for 5 min per wash, in a washing buffer. An anti-rabbit HRP-conjugated antibody (GE Healthcare) was then added at a dilution of 1:10,000 and the blots were incubated for 1 h. The blots were washed with washing buffer, and antibody binding was detected with the SuperSignal West Femto System (Thermo Fisher Scientific). The membranes were analyzed with an Amersham Imager 600 instrument (GE Healthcare Life Sciences).

### Exon Trapping Assay.

The region around VAR2 of *STAT1* was amplified by PCR from genomic DNA of a heterozygous carrier of the variant (STAT1F1: 5′-AGATGTGTTTCGTGGAGGAGA-3′, STAT1R1: 5′-CCTAAGAGGTGATACATTTAACCCT-3′). The region around *IRF7* variants (VAR3 and VAR4) was amplified by PCR from genomic DNA of heterozygous carriers of the variants (IRF7F1: GCTGTCTCCAGGG​GTCCTTG, IRF7R1: TGTTCTGGAGTTCTTTTTATTAGACTGGG.

The presence of the variants was confirmed by Sanger sequencing. Then, a 1491-nt genomic segment of *STAT1* containing exon 3 and exon 4 were amplified using primers linking the EcoRI and BamHI restriction enzymes and part of the pSPL3 vector (STAT1F2: 5′-GGGATCACCAGAATTCAGATGTGTTTCGTGGAGGAGA-3′, STAT1R2: 5′-CAGATATCTGGGATCCCCTAAGAGGTGATACATTTAACCCT-3′). Similarly, a 717-bp genomic segment of *IRF7* containing exon 10 and exon 11 was amplified using primers linking EcoRI and BamHI restriction enzymes and part of the pTAG4 vector (IRF7F2: GAATGCCTTCGAATTCGCTGTCTCCAGGGGTCCTTG,

IRF7R2: CATTGCAATTGGATCCTGTTCTGGAGTTCTTTTTATTAGACTGGG). The genomic segments were cloned into the exon trapping vector pSPL3 (STAT1) or pTAG4 (IRF7) using the In-fusion HD cloning kit (Takara) according to the manufacturer’s instructions. The VAR1 of *STAT1* was generated by site-directed mutagenesis of WT plasmid derived from the heterozygous carrier of VAR2. All constructs were confirmed to contain the correct sequence via whole plasmid sequencing (Plasmidsaurus). COS-7 cells were cultured in DMEM media containing 10% fetal bovine serum (FBS) and transfected with 1 μg of plasmid DNA (EV, WT, VAR1, VAR2) using X-tremeGENE™ 9 DNA Transfection Reagent (Sigma) according to the manufacturer’s instructions. Twenty-four hours after transfection, cells were harvested, and RNA was extracted using the RNeasy Plus Mini Kit (Qiagen). For *STAT1*, a total of 1 μg of RNA from each of the cells was reverse transcribed into cDNA using the SuperScript™ III First-Strand Synthesis System (Invitrogen) according to the manufacturer’s instructions. The cDNA was amplified by PCR with pSPL3 vector specific primers (SD6: 5′-TCTGAGTCACCTGGACAACC-3′; SA2: 5′- ATCTCAGTGGTATTTGTGAGC-3′). PCR products were analyzed by agarose gel electrophoresis and their sequences were sequenced by Plasmidsaurus linear/amplicon sequencing. For *IRF7*, RNA from samples was submitted for NextSeq for total RNA sequencing.

## Supplementary Material

Appendix 01 (PDF)

Dataset S01 (XLSX)

Dataset S02 (XLSX)

Dataset S03 (XLSX)

Dataset S04 (XLSX)

## Data Availability

AGAIN webserver, software, datasets, and documentation are publicly accessible from https://hgidsoft.rockefeller.edu/AGAIN and https://github.com/casanova-lab/AGAIN (40), under CC BY-NC-ND 4.0 License. All other data are included in the manuscript and/or supporting information.
