# Supplementary material for: Genome-wide detection of human intronic AG-gain variants located between splicing branchpoints and canonical splice acceptor sites
Source: Proc Natl Acad Sci U S A. 2023 Nov 6;120(46):e2314225120. doi: 10.1073/pnas.2314225120 (PMC10655562; doi:10.1073/pnas.2314225120)
Supplement: Supplementary file 1 — Appendix 01 (PDF) [file pnas.2314225120.sapp.pdf]

PNAS

Supplementary Information for

Genome-wide detection of human intronic AG-gain variants located between splicing branchpoints and canonical splice acceptor sites

Peng Zhang[*], Matthieu Chaldebas[#], Masato Ogishi[#], Fahd Al Qureshah[#], Khoren Ponsin, Yi Feng, Darawan Rinchai, Baptiste Milisavljevic, Ji Eun Han, Marcela Moncada-Velez, Sevgi Keles, Bernd Schröder, Peter D. Stenson, David N. Cooper, Aurélie Cobat, Bertrand Boisson, Qian Zhang, Stéphanie Boisson-Dupuis[@], Laurent Abel[@], Jean-Laurent Casanova[@,*]

[#,@] These authors contributed equally.

* To whom correspondence should be addressed:

Peng Zhang (pzhang@rockefeller.edu)

Jean-Laurent Casanova (casanova@rockefeller.edu)

This PDF file includes:

Figures S1 to S7

Other supplementary materials for this manuscript include the following:

Datasets S1 to S4

Figure S1: Eight pathogenic missplicing variants that were originally reported to disrupt branchpoints (BP). We found these variants actually served to create AG dinucleotides in the region between the BP and the downstream splice acceptor site (ACC).

BP   Variant   ACC Exon

***FAS* | g.90770494A>G**

wt: TGCTTATTTTCATATAAAATGTCCAATGTTCCAACCTACAGGATCCAGAT

mt: TGCTTATTTTCATATAAAATGTCCAGTGTTCCAACCTACAGGATCCAGAT

***FBN2* | g.127671284T>C**

wt: AAGCAGACCTGACAATGTGGTTGCAATGCTGTTTTCACAGATATTGATGA

mt: AAGCAGACCTGACAATGTGGTTGCAGTGCTGTTTTCACAGATATTGATGA

***HEXB* | g.74014605A>G**

wt: TCTAGGCCTAATAATATGTATTGCAATTTGTAACGTTAATAGCTTGCGCC

mt: TCTAGGCCTAATAATATGTATTGCAGTTTGTAACGTTAATAGCTTGCGCC

***ITGB2* | g.46321660A>C**

wt: GCCTGCCACCTCCCCAGCCCCTCCATGTGCCCTGCAGGCTTCGGGTCCTT

mt: GCCTGCCACCTCCCCAGCCCCTCCAGGTGCCCTGCAGGCTTCGGGTCCTT

***LIPC* | g.58830518A>G**

wt: GATGCCAGGCTAAGCACCGTCCCCAATCTTATATTGCAGAGCCATTTGGA

mt: GATGCCAGGCTAAGCACCGTCCCCAGTCTTATATTGCAGAGCCATTTGGA

***SLC25A20* | g.48921567A>C**

wt: CTGTAAACAGCTTCTGCTTCTGTGATTCCTTGCAGGGCATCACGGGGCTA

mt: CTGTAAACAGCTTCTGCTTCTGTGAGTCCTTGCAGGGCATCACGGGGCTA

***USH2A* | g.216040529T>C**

wt: AGTAGAAATTCATATACTTTTTTTAACAAAACAACATTTTAGGTTTACAA

mt: AGTAGAAATTCATATACTTTTTTTAGCAAAACAACATTTTAGGTTTACAA

***XPC* | g.14209889A>T**

wt: ACTATTACTGATTTTTAAAAATGCTTGTTGATAGAACTTAGTGAGCCTGT

mt: ACTATTACTGATTTTTAAAAATGCTAGTTGATAGAACTTAGTGAGCCTGT

Figure S2: Pyrimidine content and prediction of RNA stem-loop structures. (a) Pyrimidine content in three zones of interest. (b) Proportion of naturally occurring AG dinucleotides in zone 1 that could be concealed within the predicted RNA stem-loop structures. (c) Examples of RNA stem-loop structures that conceal AG dinucleotides from exposure to other base-pairing (blue arrow points in the 3' direction).

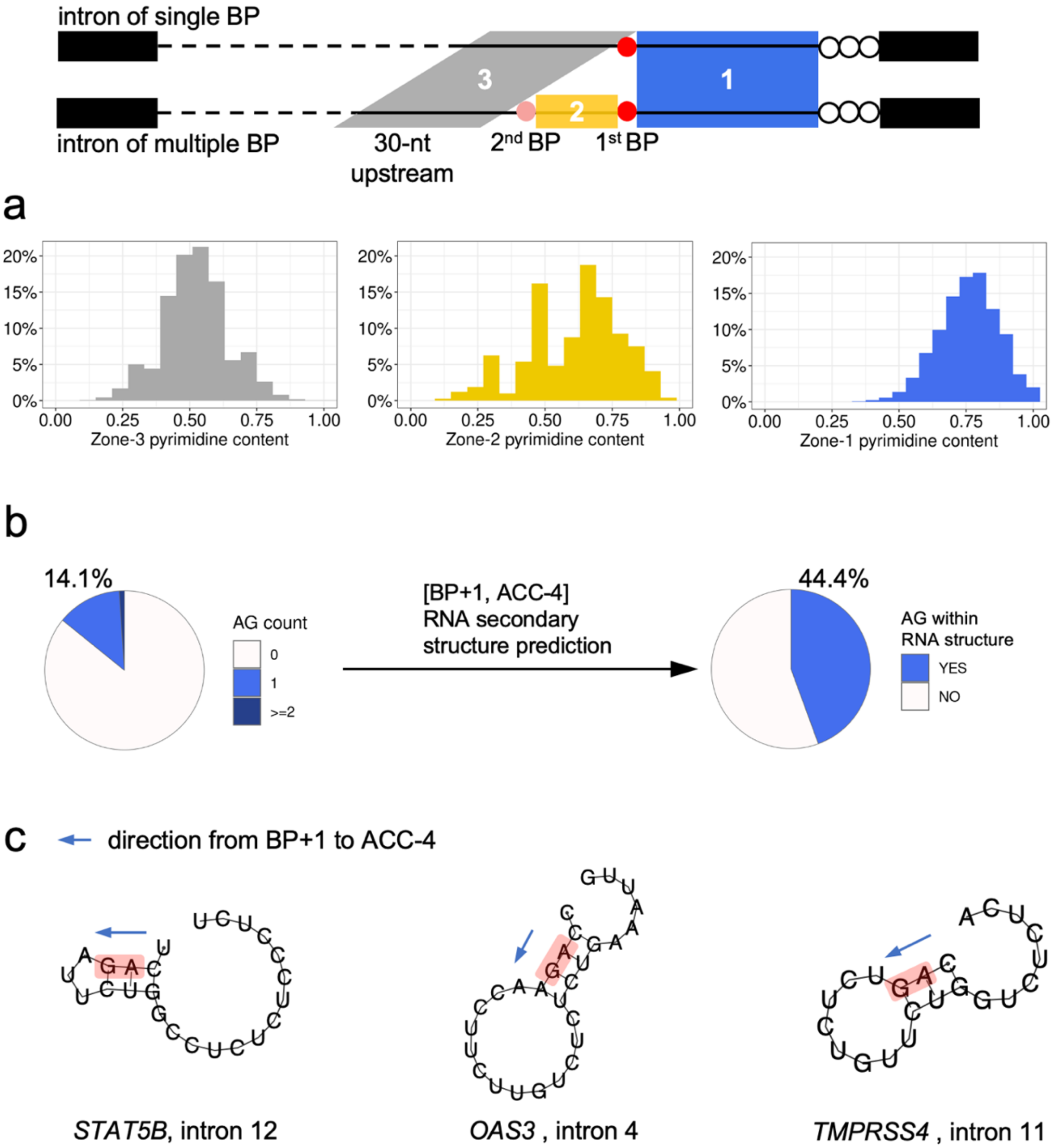

Figure S3: Schematic of AGAIN software for generating protein sequences and HGVS annotations. AGAIN focuses on the two main misspliced outcomes by AG-gain variants: new acceptor site at the newly introduced AG (NEW_ACC), and complete exon skipping (EXON_SKIP).

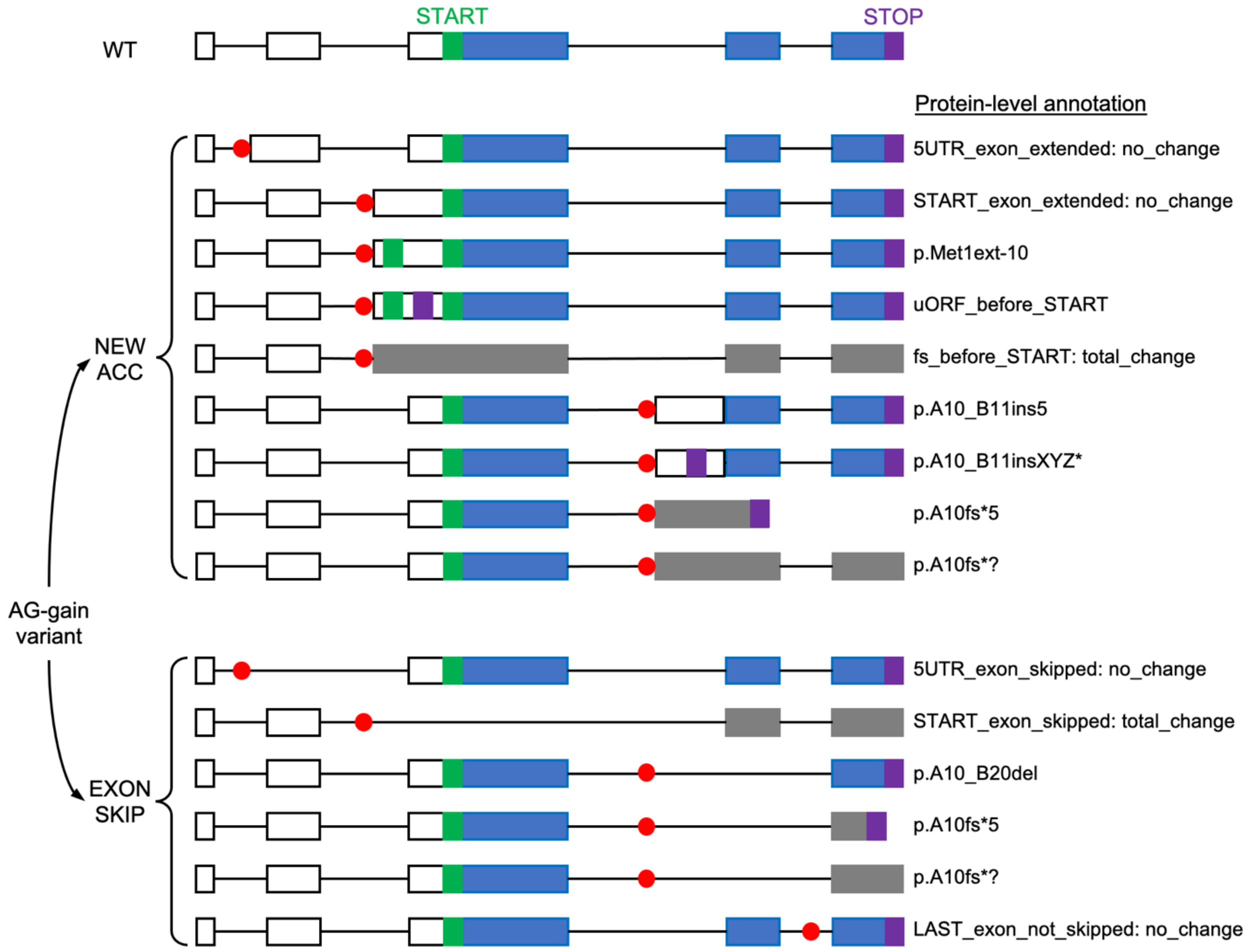

Figure S4: Analysis of 350 published pathogenic AG-gain variants in zone 1. (a) Mode of inheritance of these pathogenic intronic AG-gain variants in terms of the associated clinical phenotype (AD: autosomal dominant, AR autosomal recessive, ADAR: autosomal dominant and recessive, XLD: X-linked dominant, XLR: X-linked recessive, UKN: unknown.). (b) Missplicing outcomes and their proportions. (c/d) The distance from AG-gain variants to BP/ACC, in which the variants that created new acceptor sites are highlighted in red, and the variants that resulted in complete exon skipping are highlighted in green.

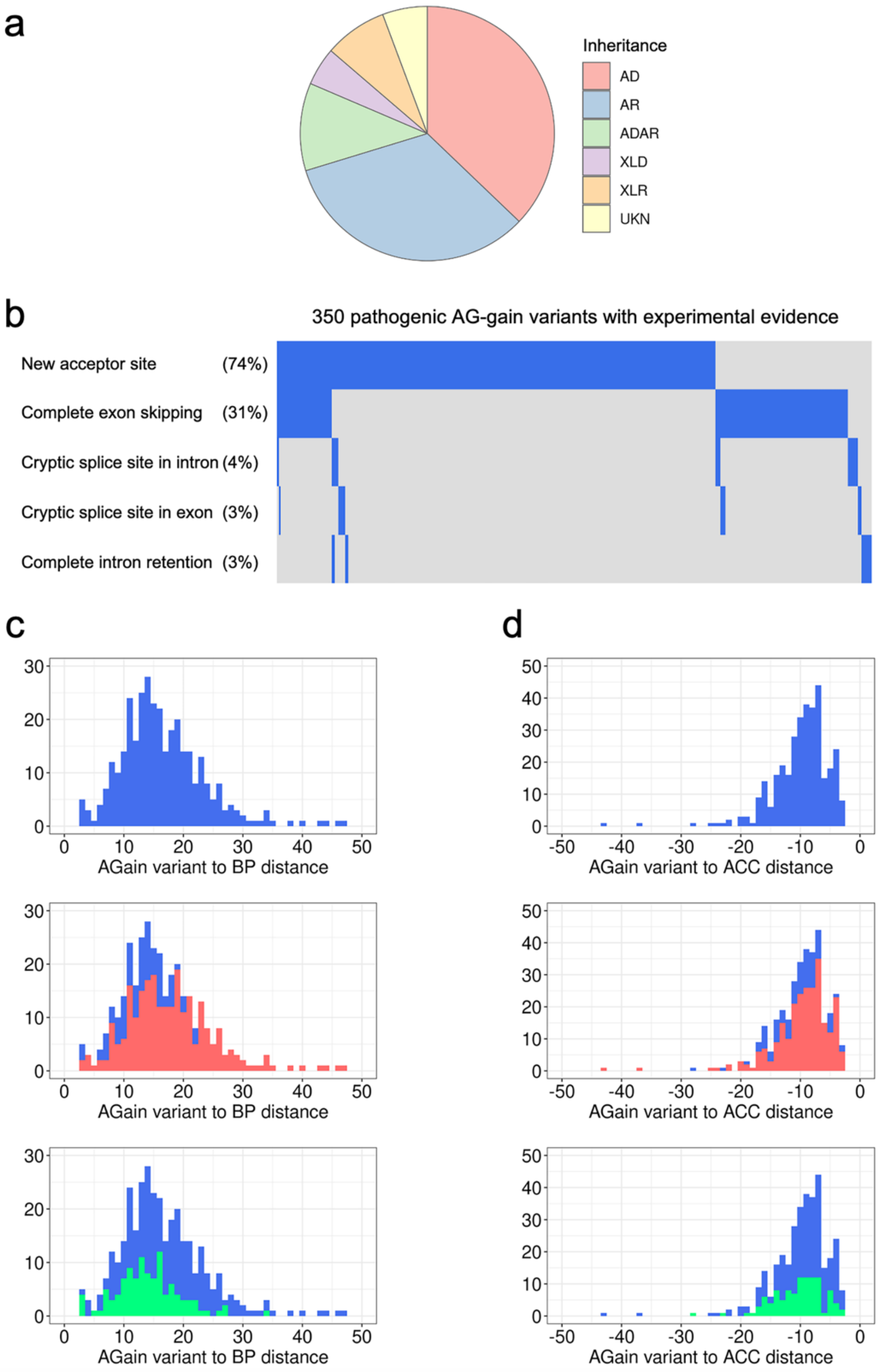

Figure S5: MAF category comparison in the human population. The proportion of six groups of intronic variant (AG-gain variants, BP variants, essential acceptor splice site variants (ACC-1 and ACC-2), all other variants in the BP-ACC region, and one million random intronic variants) in different MAF categories (singleton, rare or common), based on (a) gnomAD v2 of WES data and (b) gnomAD v3 of WGS data.

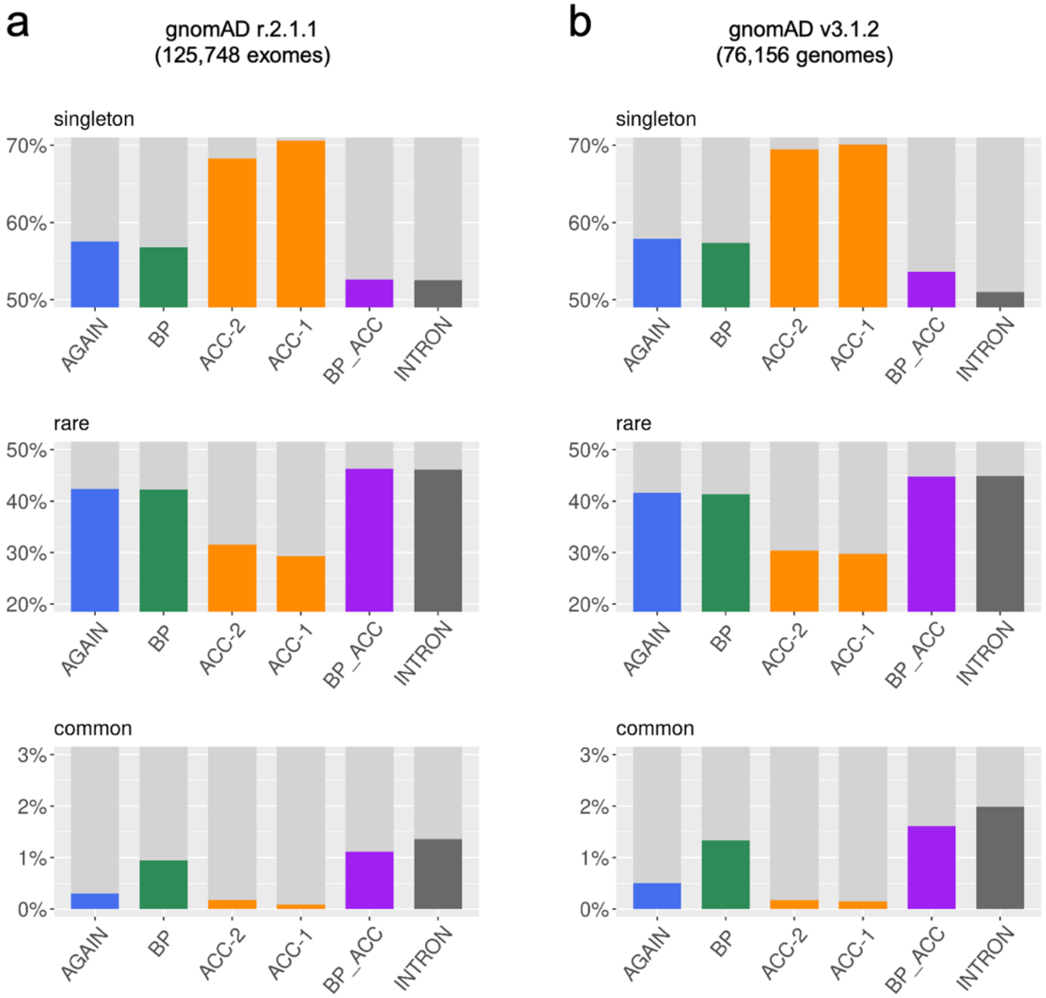

Figure S6. Normal *SPPL2A* mRNA expression levels in a patient with mycobacterial disease who carries a homozygous intronic AG-gain variant of *SPPL2A*. Total mRNA extracted from cryopreserved peripheral blood mononuclear cells from the patient (homozygous), the patient's sister (heterozygous) and two unrelated controls were used for RT-qPCR. Two different TaqMan probes for *SPPL2A* mRNA were used. Delta Ct values compared to *GUSB* (internal control) from three technical replicate reactions are shown. Bars represent the mean and standard error of the mean.

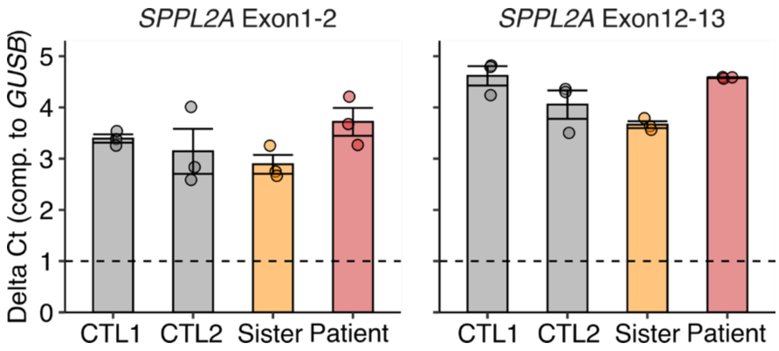

Figure S7: Genome-wide detection of 9 intronic AG-gain variants from WES data, and validation of their missplicing consequences from their paired RNA-seq data. (Green arrows indicate the locations of the AG-gain variants)

VAR	GENE	CHR	POS	REF	ALT	AGAIN YAG	AGAIN BP_DIST	AGAIN ACC_DIST	AGAIN SCORE	RNAseq Evidence
#1	*CAPN3*	chr15	42695919	C	G	YES	8	-19	5	intron retention
#2	*COL4A4*	chr2	228012304	T	C	YES	15	-3	5	new acceptor
#3	*GNPDA2*	chr4	44724274	T	C	YES	12	-14	4	exon skipping, intron retention
#4	*GPD2*	chr2	157425324	T	A	YES	12	-11	5	intron retention
#5	*KIF19*	chr17	72340351	T	A	YES	15	-9	5	exon skipping
#6	*MEGF8*	chr19	42862292	C	G	YES	13	-3	5	new acceptor
#7	*NOTCH3*	chr19	15299990	C	T	YES	15	-3	5	new acceptor
#8	*SSR1*	chr6	7310269	A	C	YES	20	-6	5	new acceptor
#9	*TBCD*	chr17	80895920	G	A	YES	13	-3	5	new acceptor

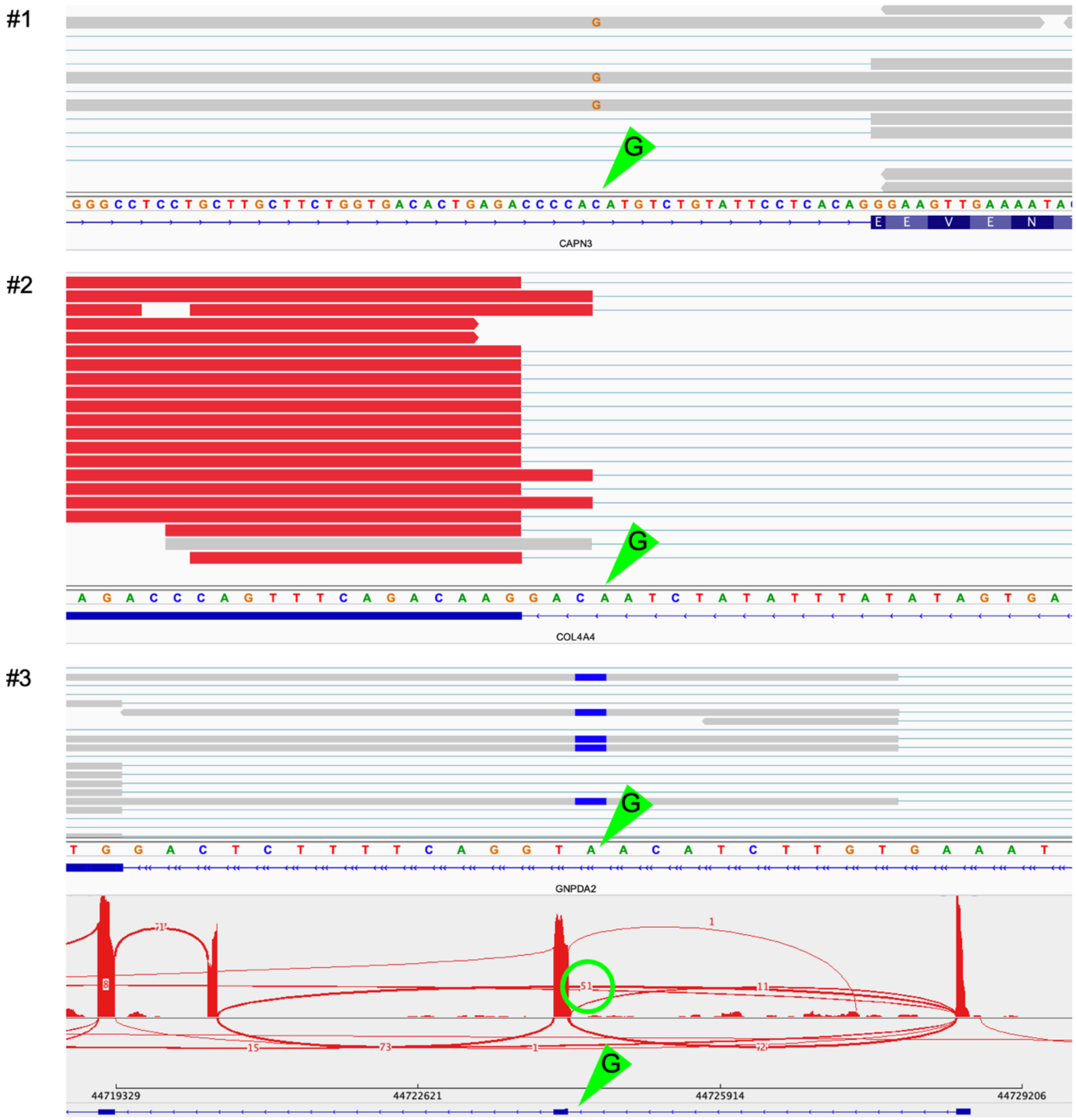

#4

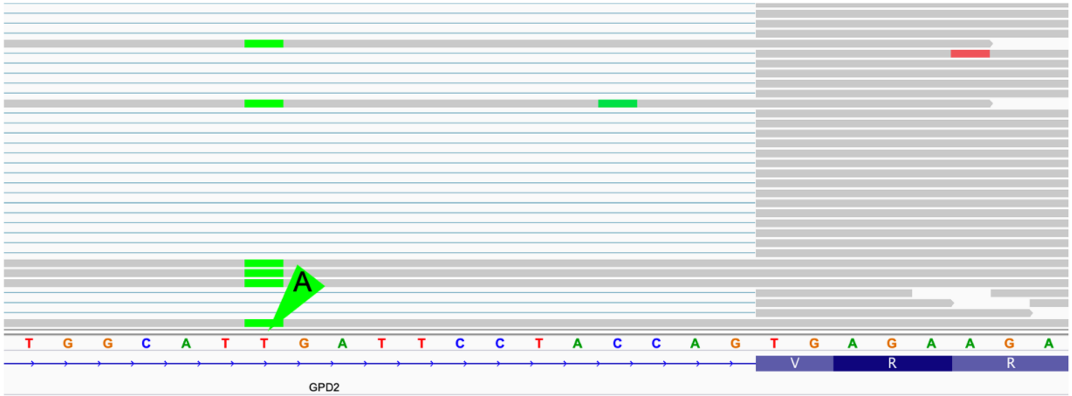
A
T G G C A T T G A T T C C T A C C A G T G A G A A G A
V R R
GPD2

#5

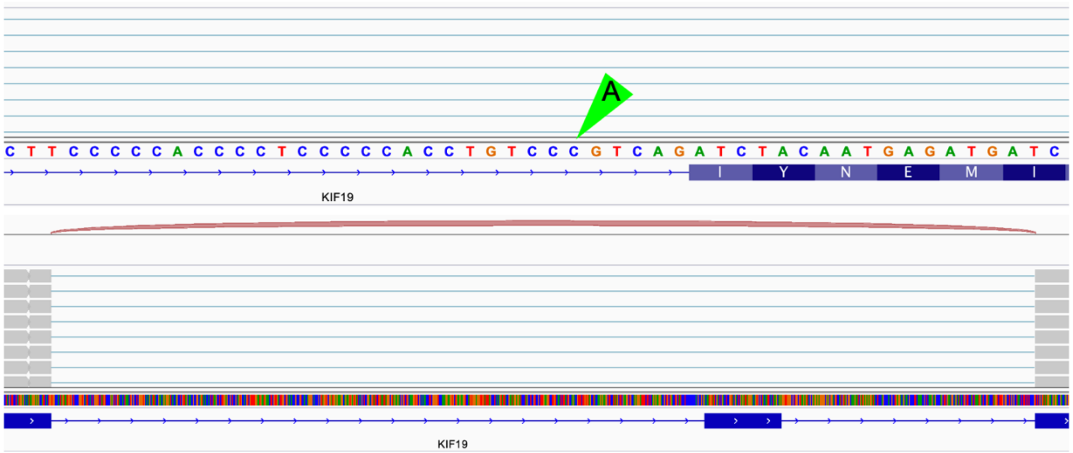
A
C T T C C C C C A C C C C T C C C C C A C C T G T C C C G T C A G A T C T A C A A T G A G A T G A T C
I Y N E M I
KIF19
KIF19

#6

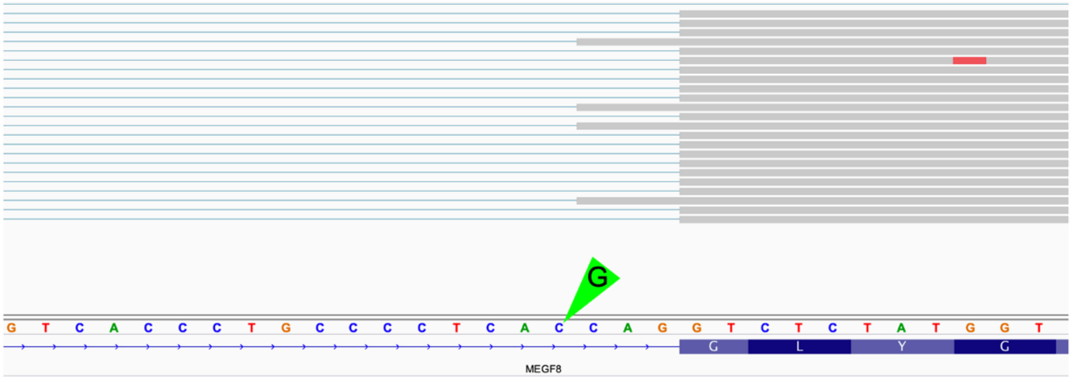
G
G T C A C C C T G C C C C T C A C C A G G T C T C T A T G G T
G L Y G
MEGF8

#7

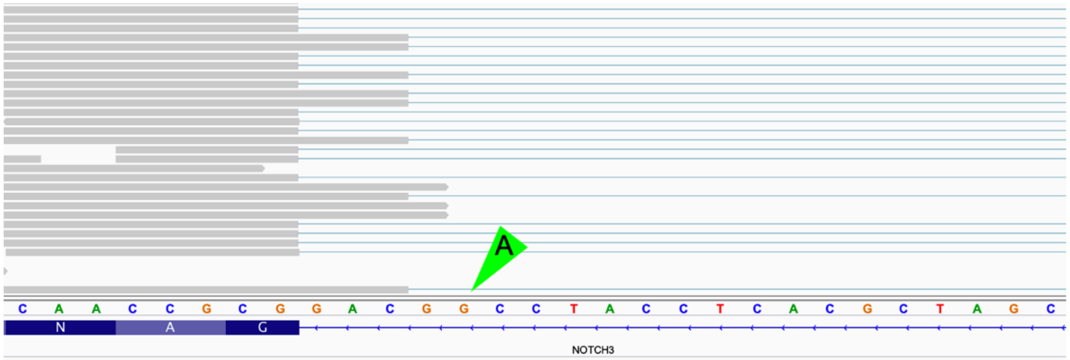
A
C A A C C G C G G A C G G C C T A C C T C A C G C T A G C
N A G
NOTCH3

#8

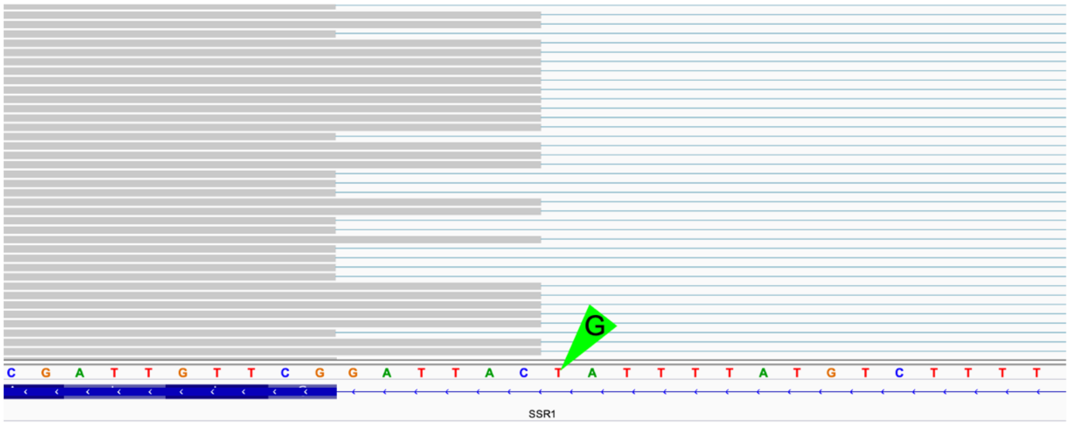
G
C G A T T G T T C G G A T T A C T A T T T T A T G T C T T T
SSR1

#9

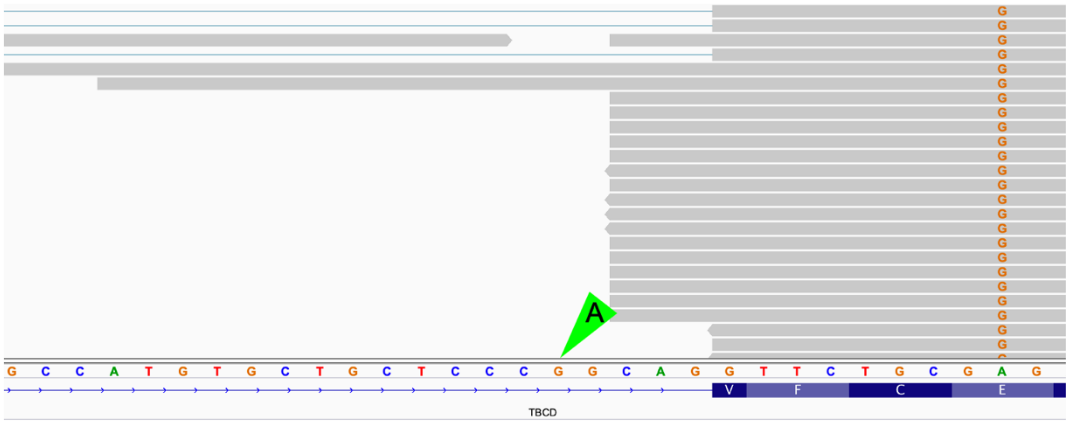
A
G C C A T G T G C T G C T C C C G G C A G G T T C T G C G A G
V F C E
TBCD
